# Mitigating housing market shocks: an agent-based reinforcement learning approach with implications for real-time decision support

**DOI:** 10.1080/17477778.2024.2375446

**Published:** 2024-07-09

**Authors:** Sedar Olmez, Alison Heppenstall, Jiaqi Ge, Corinna Elsenbroich, Dan Birks

**Affiliations:** a School of Geography, University of Leeds, Leeds, UK; b The Alan Turing Institute, British Library, London, UK; c School of Social Political Sciences, University of Glasgow, Glasgow, Scotland; d School of Health and Wellbeing, University of Glasgow, Glasgow, Scotland; e School of Law, University of Leeds, Leeds, UK

**Keywords:** UK housing market, agent-based model, reinforcement learning, crisis management, decision-making

## Abstract

Research in modelling housing market dynamics using agent-based models (ABMs) has grown due to the rise of accessible individual-level data. This research involves forecasting house prices, analysing urban regeneration, and the impact of economic shocks. There is a trend towards using machine learning (ML) algorithms to enhance ABM decision-making frameworks. This study investigates exogenous shocks to the UK housing market and integrates reinforcement learning (RL) to adapt housing market dynamics in an ABM. Results show agents can learn real-time trends and make decisions to manage shocks, achieving goals like adjusting the median house price without pre-determined rules. This model is transferable to other housing markets with similar complexities. The RL agent adjusts mortgage interest rates based on market conditions. Importantly, our model shows how a central bank agent learned conservative behaviours in sensitive scenarios, aligning with a 2009 study, demonstrating emergent behavioural patterns.

## 1. Introduction

^1^Agent-based models (ABMs) have been adopted in various research areas since their inception in the late ‘90s to early 2000s (Filatova, 2015; Ge, 2017; Groff Elizabeth et al., 2018; Heppenstall et al., 2006; Kothari et al., 2014; Tang & Bennett, 2010). ABMs enable researchers to simulate a complex system with autonomous agents interacting with each other within an environment. The main strength of ABMs over mathematical models is that they simulate, validate, and verify behavioural characteristics at granular spatio-temporal resolutions (Olmez, Thompson, et al., 2022; Secchi, 2015; Todd et al., 2017). This allows researchers to analyse complexity and investigate how a studied phenomenon develops at the individual level (Epstein & Axtell, 1997). This article focuses on housing markets and investigates market shocks, which are unanticipated changes to economic variables that impact the market’s health (Ramey, 2016).

ABM has been used in housing market research. Researchers investigated the emergence of housing bubbles (Axtell, 2014; Erlingsson et al., 2014; Ge, 2014, 2017), the dynamics of urban regeneration (Jordan et al., 2011, 2012; Picascia et al., 2014), and how real-world shocks such as the 2008 financial crash affected the housing market (Gilbert et al., 2009; Hamill & Gilbert, 2015). The number of ABMs for studying housing and financial markets is growing (Bae et al., 2019; Baptista et al., 2016; Carstensen, 2015; Geanakoplos et al., 2012). These models generally allow agents to make decisions in volatile scenarios, either to hedge against volatility or profit from it (Fischer & Riedler, 2014, Todd et al., 2017; Westerhoff, 2010).

A research area less explored is applications of machine learning (ML) algorithms supporting decision-making in alleviating shocks once they have occurred, which central-bank policymakers can use to inform policy. Most models cited earlier examined how, when, and why shocks occur. However, developing techniques to counteract these shocks can reduce the impact on the economy and people’s health (Oguibenine, 2011). This article proposes a hybrid model that integrates reinforcement learning (RL), with a housing market ABM. Conducting a series of experiments, we investigate if an intelligent adaptive central bank agent (Almahamid & Grolinger, 2021; Littman, 2015; Mehta, 2020) can learn trends from a housing market in real-time. During learning, this central bank agent makes decisions to fulfil a goal, for example, decreasing homelessness. In this article, “intelligent adaptive agent” is defined as: “systems or machines that utilise inferential or complex computational algorithms to modify or change control parameters, knowledge-bases, problem-solving methodologies, course of actions, or other objects in order to accomplish a set of tasks required by the user” (Imam & Kerschberg, 1997).

We identified several benefits of utilising RL in the housing market domain (1) researchers can test macroeconomic policies in a safe “sandbox” environment without real-world consequences. (2) researchers can adopt various RL goal criteria to test policy interventions in the housing market. (3) researchers can test various interventions in their housing markets and document the steps to counteract these interventions. (4) shocks (crashes) can artificially be induced to speed up learning, whereas market shocks are rare events in the real world.

This article replicates an ABM of the UK housing market (Gilbert et al., 2009). Other notable housing market ABMs exist (Baptista et al., 2016; Filatova, 2015; Ge, 2017; Rosenfield et al., 2013; Yun & Moon, 2020). However, we found that either these articles were not open access and did not include download links to the models (Filatova, 2015; Ge, 2017) or the articles were open access. However, no documentation was provided to access the ABMs (Baptista et al., 2016; Rosenfield et al., 2013; Yun & Moon, 2020). We chose (Gilbert et al., 2009) as it was well received by researchers (61 citations as of May 20 2022 on Google Scholar), for its documentation. Furthermore, (Gilbert et al., 2009), strikes a good balance between simplicity (where results are tractable) and realism (simulating important processes unique to the UK housing market, such as chain trade and can replicate empirical patterns).

To investigate whether RL can manipulate the housing market, this article reproduces two identical experiments conducted in (Gilbert et al., 2009) as a comparator. Where exogenous shock events occur, and the decisions made by the central bank RL agent are observed. These results are compared to baseline scenarios where the RL intervention is removed. The model outputs reflect the consequences of RL decisions, and findings are compared with the original assertions made in (Gilbert et al., 2009).

The motivation behind the investigation lies in the potential applications of RL for policymakers. By assessing the adaptability and decision-making prowess of the RL agent, particularly in adjusting mortgage interest rates based on market conditions, our study offers insights into novel strategies for mitigating the consequences of unforeseen events in the housing market. These insights aim to empower policymakers with a proactive tool-set, allowing them to navigate and respond effectively to the challenges posed by housing market dynamics.

To summarise, this article will investigate whether (i) an RL agent can be integrated with a housing market ABM and (ii) can an RL agent be trained using input data from the housing market ABM and make decisions to counteract shocks when they occur during run-time.

Section 2 reviews pre-existing studies with Section 3 describing the ABM developed for this article, including the RL application of the central bank agent. The results Section 5 defines the experiments conducted and the subsequent outcomes. Lastly, a discussion and conclusion Section 6 discusses the findings from the experiments, limitations and strengths and concludes with future avenues to be explored.

## 2. Literature review

Economic crises sometimes take the form of debt crisis (where a government’s debt increases while repayments decrease), banking crisis (when a large swathe of people withdraw their savings as confidence in the banks depletes), asset bubble burst (i.e., housing bubble bursts which leads to a sudden devaluation of houses, an example of this was the subprime mortgage credit crisis in 2007–2008 (Dou & Wang, 2014)) and balance of payment crisis (when a country cannot afford the price of imports or services). Regulatory policy is vital when a country tries to prevent or counteract an economic crisis (Malyshev, 2015), such as a central bank’s monetary policy. Martin et al. (2022, p. 3) researched whether central banks can stabilise housing markets via interest rates. Researchers found that the ability of central banks to manage housing markets by increasing interest rates, which softens the demand pressure on house prices, is limited. However, they note that “central banks can significantly improve the stability of housing markets by dynamically adjusting interest rates”. Researchers agree that ML can be used to support decision-makers in alleviating economic crises (Chiriţă, 2011; Ho, 2020; Loukis et al., 2020; Maghdid & Ghafoor, 2020; Nik et al., 2016).

RL algorithms are a subset of ML approaches which enable artificial agents to learn. An agent tries to complete a task and, in doing so, maximises its internal rewards (Sutton & Barto, 2018). Typically, these agents learn how to complete a task through trial-and-error by interacting with their environment (Kaelbling et al., 1996). RL theory was derived from empirical observations of the psychological and neuroscientific studies in animal behaviours (Mnih et al., 2016). RL has successfully demonstrated the ability of an agent to learn how to achieve long or short-term goals through interactions with the immediate environment, the reflection of one’s past knowledge and decisions influenced by rewards and penalties. Many applications of RL exist, including but not limited to (Liu et al., 2020) where researchers optimise the choice of medications identifying the correct drug dosing and timing of interventions. Spatharis et al. (2019) developed a model where air traffic is managed through an RL agent that observes millions of data points and makes optimal decisions as to when and where planes should land.

Most RL applications in the housing market domain are related to “house price forecasting” and prediction techniques (Chen et al., 2017; Zhan et al., 2020). Some studies have integrated deep neural networks to investigate housing markets, given the recent growth in data from websites like Craigslist, Rightmove, and Gumtree. Researchers trained a neural network using textual data to identify how the rental market dynamics were changing (Zhou et al., 2019). Similarly, researchers implemented neural networks, to classify physical and socio-demographic characteristics, to assess how interrelated these factors are in the housing market of Budapest, Hungary (Norwegian, 2007). An article developed an early warning system that identified market volatility from house price training data (Park & Ryu, 2021). A drawback of this approach was that rich data sources are usually placed behind paywalls, and the neural network would have to be trained every time new data was accessible. In our research, the ABM of the housing market acts as a continuous data stream. Most importantly, in our approach, we can artificially introduce shocks (crashes) to the system to speed up learning, whereas market shocks are rare events in the real world.

Researcher articles such as Yamaguchi et al. (2018) show how RL identifies specific behaviours worms possess pre and post-feeding. Sali et al. (2021) used RL to deal with the feature selection problem, where researchers identified the most accurate and optimal features for reducing computation costs. As evidenced by the limited yet critical studies above, RL can learn to identify a particular phenomenon/pattern in data and develop effective interventions using neural networks to achieve a particular goal. Such as identifying the correct dosage for a patient’s medication (Jalalimanesh et al., 2017). Compared to the above studies, examples of applied ABM and RL in housing market research are rare (only four articles with the terms “housing market”, “reinforcement learning”, and “agent-based”, source Web of Science). The articles (Cincotti et al., 2005; Suzuki et al., 2014; Zhou, Wu, et al., 2017) utilise RL as an optimisation method to identify the most efficient strategies in power-to-power (P2P) sharing of energy between households and companies. Kang et al. (2019), on the other hand, uses data assimilation and RL to fit real-world Korean housing market data to an ABM. In the light of these advances, this article contributes to the literature by integrating an RL decision-making algorithm in a housing market ABM focusing on shocks. It is worth noting that the work proposed here is purely experimental at this stage and acts as a proof of concept.

In this article, the artificial “central bank” agent observes data streams from the housing market ABM (Olmez, 2022) and makes dynamic decisions that impact the market (such as raising, holding or reducing interest rates), demonstrating how RL can be used to stabilise a market effectively in real-time in simulation. The opportunities for using RL and ABM are considerable. For example, this article demonstrates how RL can support decision-making in stabilising the housing market during volatile times. However, in future studies, it may be used to identify early signals of a recession or a financial crisis and alleviate the negative impact of exogenous shocks such as pandemics.

## 3. The housing market model

**Figure 1.**
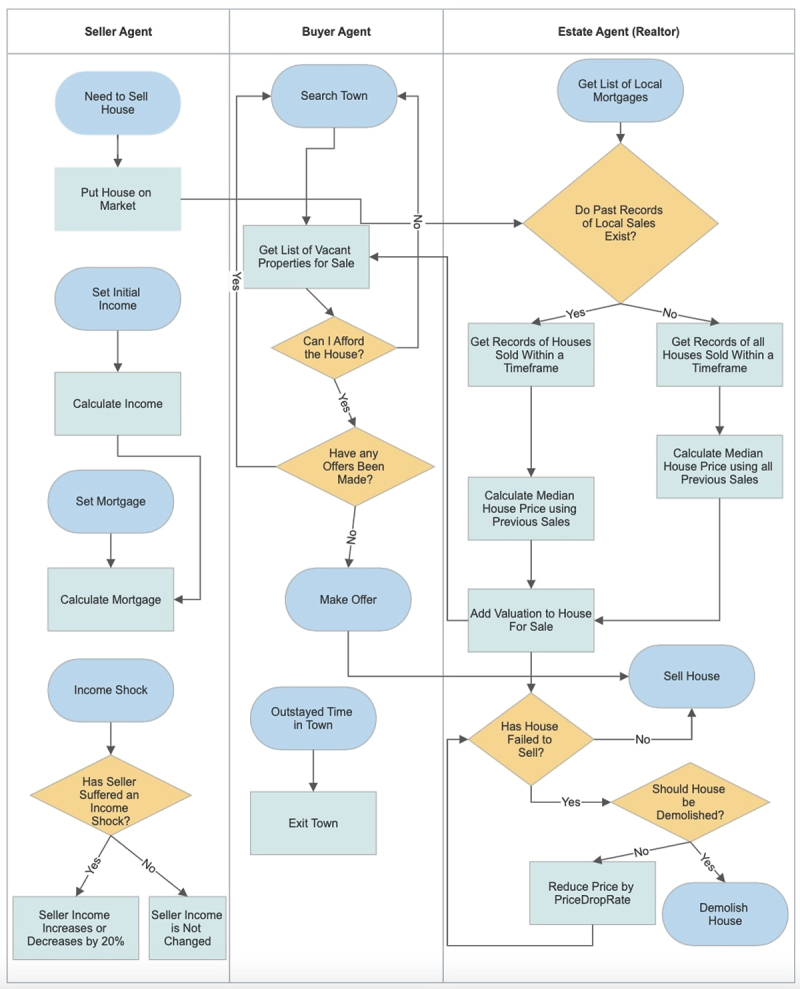
Flowchart presenting decisions the Seller, Buyer and Realtor (Estate) agents undertake.

The housing-market model simulates the characteristics of the UK housing market. The model contains agents that are either buyers, sellers, estate agents or houses. An aggregate distribution of these agents interact in the environment where agent-environment and agent-agent interactions grow micro and macro emergent properties. The model simulates the interactions between buyers and sellers, who utilise information from local estate agents Figure 1. Buyers make offers depending on budget and successful acquisition of mortgages, while sellers depend on valuations from estate agents, who evaluate a property’s price depending on past sales and a markup.

**Figure 2.**
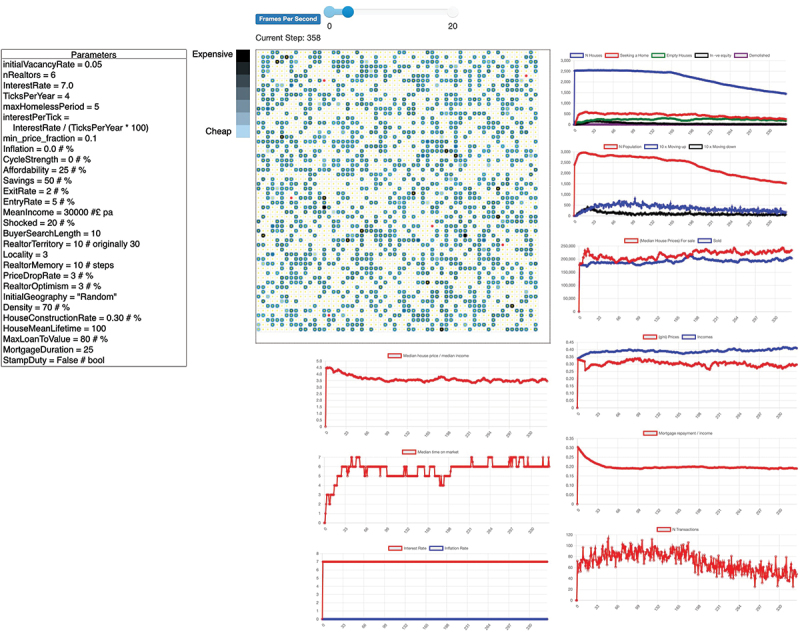
The user interface of the model, the parameters that can be changed on the left, the visual representation of the ABM in the centre where the small squares represent houses. Yellow dots are occupants, red dots are estate agents, white grid cells represent free space. Output plots are on the right.

The proposed ABM in Figure 2 is a reproduced version of (Gilbert et al., 2009). The purpose of this reproduction in the Python programming language (Olmez, 2022) was to access a broader set of novel ML algorithms and tools which the Netlogo framework was not able to harness. The model described in the following paragraphs was inspired by the works from (Gilbert et al., 2009).

### 3.1. Model environment

The environment generates a 60 × 60 grid, which can be changed depending on computing power – producing 3600 cells that can either be a house, occupied house, unoccupied space or an estate agent. Houses are randomly distributed and, depending on density, in the case of Figure 2 70% of the space is occupied. The initialVacancyRate sets the proportion of unoccupied houses at the start, making these houses available to buy. The price of these unoccupied houses follows the same rules outlined earlier. Estate agents find the highest valuation from previous sale records. The house prices are randomly distributed using a uniform random distribution, as is the case in Figure 2. Each house has a quality index calculated upon initialisation. This measure is a ratio of the average price of other houses within the locality of the constructed house’s sale price. The process mentioned above adheres to Tobler’s first law of geography (Miller, 2004) which states that nearer things are more likely to be similar than those farther apart. Every output parameter is described in Appendix A, Table A1.

### 3.2. Seller agents

Every step, the model moves forward in time; a step is 3 months defined by the TicksPerYear parameter Figure 2. A percentage of homeowners (ExitRate parameter) vacate and try to sell the house at a price set by the estate agent valuation. If the house does not sell at the current timestep, it remains on the market for the next period. Every homeowner agent has an initial income determined randomly using a gamma distribution from parameters 1.3 and $5\times 10^{-5}$ multiplied by the MeanIncome parameter. Furthermore, a mortgage is calculated by the ratio of the Affordability parameter, divided by interestPerTick multiplied by the owner’s income. Initially, the mortgage duration is 25 years. However, this can be changed. People borrowing money must have some deposit from their capital determined by MaxLoanToValue parameter and their mortgage. At every step, a percentage of homeowners suffer income shocks determined by the Shocked parameter, which is **+20%**, and the same percentage suffers a shock of −20%. This leads to some homeowners having their income increase or decrease by this percentage permanently. When the ratio of the mortgage repayment is higher than twice the affordability, the homeowner trades down. Conversely, they trade up when the ratio is less than half the affordability.

### 3.3. Estate agents

The term estate agents is used interchangeably with realtors. Every realtor agent has a coverage radius called the RealtorTerritory. Any house outside a realtor’s territory is assigned the closest realtor calculated by the Euclidean distance. Each realtor keeps records of the previous sale. These records contain the following information: record ID, the house sold, selling price and date of sale. At the start, the mortgage value of each house is sent to one local realtor, providing realtors with a starting point for their valuations (Gilbert et al., 2009). When a seller asks for a valuation, the realtor looks through their records within the last RealtorMemory timesteps and gathers all the house prices of houses sold locally multiplied by the quality index of these houses. It then calculates the median house price of these previous sales as a valuation. If no sales have been made within the locality and period, any past sales made within the locality are considered regardless of time. Every valuation made is increased by the RealtorOptimism percentage, allowing realtors to try to sell a house more than the going rate. Lastly, if a house fails to sell at timestep N, the selling price of this house is reduced by PriceDropRate %, and it remains on the market for N + 1 until it is sold or demolished.

### 3.4. Buyer agents

At each timestep, people arrive in town. The amount depends on the EntryRate parameter, which is a percentage of the current population Figure 2. New entrants and sellers who remain search the whole town for several timesteps defined by BuyerSearchLength parameter. Looking for vacant properties for sale that they can afford and which no offers have yet been made. Any accumulated capital from buying and selling can be put towards the costs of the new property. Subsequently, buyers choose the nearest property in price to their maximum budget and make an offer at a price set by the seller. The first buyer to make an offer has their offer accepted.

### 3.5. Sales

A sale is only successful if the chain of buyers and sellers remains intact. A successful chain can only occur if the house being bought is either empty or the seller succeeds in purchasing a new house and moving to that house. The people leaving town move out, and potential buyers move into these vacant properties if their offers are successful. Once all sales down the chain are complete, the model moves forward one step in time. When a house is sold, the seller receives the sale price and uses as much of it as necessary to pay off any remaining mortgage. If money is in excess, this is added to capital and can be used as a partial or complete payment of the house being bought. Conversely, if the sale price is less than the amount remaining to pay off the mortgage, the seller is in negative equity and withdraws the house from the market. The estate agent records successful sales and uses these records (as discussed above) to value houses within the same area. Finally, if an offer falls through, it lapses (Gilbert et al., 2009).

### 3.6. Building new houses

New houses are constructed at random empty grid cells at every timestep. The number of houses depends on the HouseConstructionRate Figure 2 % of the total number of constructed houses unless there are no empty cells.

### 3.7. Demolition of houses

Every house has a lifetime set when it is created. This is drawn from a random exponential distribution with a mean of HouseMeanLifeTime. When a house reaches its lifetime, it is demolished, and the cell becomes vacant and available for new construction. If a house’s sale price falls below one-tenth of the median price of all houses, it is demolished. If someone occupies a house that is being demolished, they attempt to purchase a new home, and if they fail after MaxHomelessPeriod, they leave town.

### 3.8. Model outputs

The data produced from the model are presented below. Visually, houses are assigned a colour that reflects their current value. The lighter the shade, the cheaper the house and vice versa Figure 2. The quantitative model outputs are the following:

- Number of houses, empty houses, and demolished houses. Number of people searching for a home, the number of people occupying a home in negative equity, and the number of transactions.
- The number of people in the model.
- The median house price of houses for sale and sold.
- The Gini index of the median house prices and median incomes.
- The ratios between median house price to median income, and mortgage repayments to median income.
- The mortgage interest rate, inflation rate and median time houses have spent on the market.

### 3.9. Reinforcement learning agent

RL allows agents to learn without explicitly telling the agent what the task is or how it is completed. A feedback reward allows the agent to learn through trial-and-error by performing actions for each state in the environment. If the reward is positive, the agent has enacted a desirable action. If the reward is negative, the action is undesirable (Sutton & Barto, 2018).

Given how well policy-gradient methods have performed (Agarwal et al., 2020; Schulze et al., 2017), this was an applicable approach. Put simply, we denote a policy as π, where πθ(a|s) is the probability of taking action a in state s and θ are the parameters of our policy. Our goal is to update $\theta_{t}$ to $\theta_{t+1}$ such that we reach the optimal policy. In our model, the optimal policy would be the state where the “healthy housing market” criteria (described below) are met. If we assume a∗ is the optimal action, i.e., raise interest rates by 0.01 at time t, then we want to perform gradient ascent on πθ(a∗|s) (ascent as we want to increase our cumulative reward). Therefore, at each iteration, we update θ in the following way $\theta_{t+1}=\theta_{t}+\alpha \nabla \pi_{\theta_{t}}(a*|s)$ this can be described as we keep “pushing” towards more of action a∗ in our policy, which is indeed what we want as raising the interest rate by 0.01 will mean we are closer to achieving our “healthy housing market” criteria.

**Figure 3.**
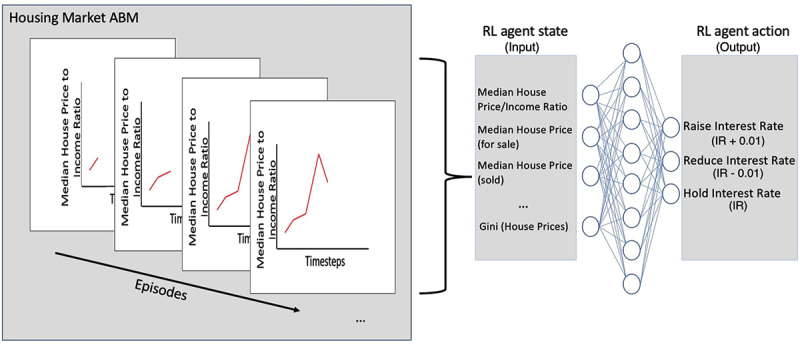
RL central bank agent neural network, that determines the central bank agent’s decision regarding interest rates, in the current instance, an action with high probability may be to raise interest rates as a sharp increase in house price to income ratio is observed.

This article proposes a novel application of RL to identify and counteract market shocks in the housing market in real-time in simulation. Several steps were taken to integrate RL with the housing market ABM:

1. Re-producing the well-known housing market (Gilbert et al., 2009) in a new framework (Olmez, 2022) to use as our experimental sandbox.
2. Replicating two experiments originally described and subsequently investigated in (Gilbert et al., 2009, p. 5) known as the loan-to-value experiments where, the MaxLoanToValue parameter is set to 80% and 100% respectively. During these experiments, an exogenous shock known as “ratefall” where a sharp increase 7% to 10% in interest rates is triggered. These two experiments were selected, as in the original article, Gilbert et al. demonstrated how the impact varying interest rates on the market were prone to being less sensitive when loan-to-value was reduced compared to loan-to-value being 100%. These experiments are poised to test RL’s ability to adapt its behaviour in two similar initial conditions but with very different outputs.
3. Training the RL agent on the housing market scenarios over 100 episodes, this process can be observed in Figure 3.

Due to computational complexity of training RL agents with neural networks (Baker et al., 2019; Juliani et al., 2018; Olmez, Birks, et al., 2022; Sutton & Barto, 2018), the experiments are kept concise. It is worth noting that the proposed model is a proof-of-concept used to demonstrate how the adaptive qualities of cognitive models such as RL are suited to modelling housing market dynamics and supporting decision-making to counteract shock events.

This article models simplistic behaviours of a central bank agent. As discussed in Section 2. In reality, a central bank has more policy tools and goals to achieve beyond housing market stability. This simplicity is necessary to demonstrate a proof-of-concept. In future research, these behaviours will become more advanced.

To train the RL agent, we first identify the healthy housing market indicators. This way, the agent can learn to differentiate between an undesirable state and a desirable one. A healthy housing market is characterised by stability, affordability, and accessibility Maliene2008SustainableRelations (Cai & Lu, 2015; Maliene et al., 2008)., Stability is indicated by small fluctuations in house prices, suggesting a balance between supply and demand (Zhu, Betzinger, et al., 2017). Affordability is often affected by the house price to income ratio, with a lower ratio reflecting better affordability for the average household. Accessibility can be indirectly measured through the prevalence of negative equity, as excessive negative equity rates can imply barriers to entry or exit from the housing market.

In line with these principles, we have set the following indicators to define a healthy housing market within our model:

- Stable median house prices for sale with small fluctuations up to ≤400,000.
- Median house price to income ratio ≤7.
- Number of people in negative equity is ≤5% (123) where N_people=2466.

If the above conditions are met, we have a desirable state (reward returned to the central bank agent, 0). If all but one of these conditions are not met, we are in an undesirable state (reward returned, −1).

The results from the RL process are presented and compared to base case scenarios in the following section. The RL outputs illustrate the central bank agent’s learning process during training and how the RL agent adapts to the LTV scenarios. We compare findings to those discussed in the original article to demonstrate how RL has or has not benefitted the housing market in alleviating shocks and fulfilling goal criteria.

### 3.10. Training methodology

The RL agent was trained using an offline training methodology, as this allows for controlled and repeatable experiments essential for rigorous scientific evaluation. Training involved the following steps:

1. The agent observed state-action pairs from a pre-simulated dataset generated by the ABM representing the housing market dynamics over multiple historical scenarios.
2. Based on the feedback (rewards) calculated from these scenarios, the agent learned optimal policies using the Proximal Policy Optimisation (PPO) algorithm, aimed at maximising long-term rewards.
3. The training phase consisted of 100 episodes, with each episode representing a complete simulation run from start to finish of the housing market model.
4. Convergence of the agent’s learning was determined by a consistent increase in rewards and stabilisation of policy outputs across episodes.

Post-training, the agent was tested in a simulated environment that replicated emerging housing market trends to evaluate its real-time decision-making capabilities.

## 4. Model validation

The proposed model (Olmez, 2022) was reproduced by interpreting (Gilbert et al., 2009) source code, refer to Appendix B, Figure B1 for class diagram. The behaviour of our model must be compared and deemed similar to the original, whereby model outputs produce similar trends in data. If the model outputs differ, we have deviated from the original at some point in the development. Suppose the model produces similar trends in the data outputs. We can be confident in our model’s behaviour in producing realistic trends of the UK housing market. Model replication is an important topic in the ABM literature, as discussed by (Donkin et al., 2017, p. 1) “model replication remains rare, yet is vital to assessing the repeatability of existing ABMs”.

Two housing market scenarios were simulated for both models using input parameters in Appendix A, Table A2. In scenario one, no shock is introduced, and in scenario two, a shock is introduced mid-simulation run. This allows us to compare behaviours in two unrelated scenarios to quantify two completely different outcomes.

To compare both models, we adopt a visual statistical approach known as quantile-quantile (Q-Q) plot, the benefits of which have been thoroughly discussed in the following literature (Dhar et al., 2014; Oldford, 2016). Where, two probability distributions are compared using their quantiles. In our case, one variable in (Olmez, 2022) is compared to the same variable in (Gilbert et al., 2009). Furthermore, over 100 model runs over 100 simulation years are drawn for each scenario providing a large sample size to quantify the stochasticity produced and output-variability (Bogdoll et al., 2012; Lelei & McCalla, 2019). A one-degree gradient (45${\;}^{\circ }$) reference line is plotted to compare variables. If x=y, each point sits on the reference line, then both variables compared are identical and vice versa.

Due to the large volume of output variables (18) Figure 2. We select a sub-set of Q-Q plots to use in the results. These include parameters that capture the housing market’s health, for example, the median house price to income ratio and the median price of houses for sale.

**Figure 4.**
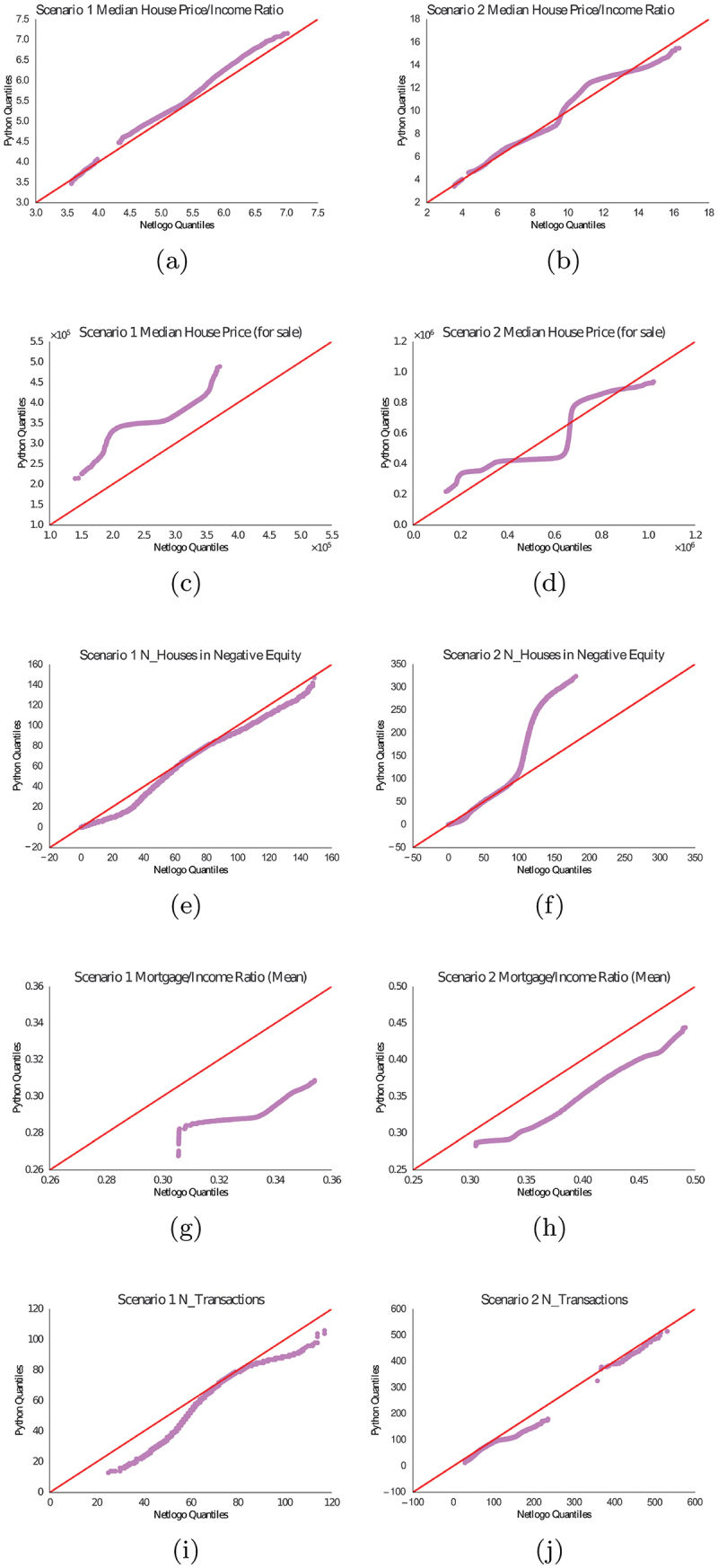
Q-Q plots comparing the distributions of model output variables. The solid line indicates x=y for reference. Where (a-b): Median house price to income ratio (Scenarios 1–2), (c-d): Median house price for sale (Scenarios 1–2), (e-f): Number of households in negative equity (Scenarios 1–2), (g-h): Mean mortgage to income ratio (Scenarios 1–2), (i-j): Number of transactions (Scenarios 1–2).

Figure 4 presents (Gilbert et al., 2009) on the x-axis and (Olmez, 2022) on the y-axis. Each column and row represents the scenario and variable respectively. These results show a normal distribution and results are correlated which quantitatively replicate similar trends, that sometimes deviate due to stochasticity, such as (Olmez, 2022) overestimating (Figures 4(c,f)) or underestimating (Figures 4(g,h)). Note, due to model architectures, frameworks and other factors, models cannot be replicated perfectly as highlighted by (Donkin et al., 2017; Yingfei, 2009).

The descriptive statistics Appendix B, Figure B2 show that 79% of the output variables returned a positively correlated Pearson’s correlation coefficient, where 11 of these from scenario two had r>0.60 which were all statistically significant p<0.01. In scenario one, eight variables had r>0.60 and p<0.01.

In scenario two, all variables returned an 0.659≤r≤0.992, all statistically significant, Figure 4. In scenario one, 0.235≤r≤0.839 four variables were statistically significant. The only variable that was not statistically significant was the mean mortgage-to-income ratio for scenario one. The most likely reason could be stochasticity, where the algorithm has more steps to process (Donkin et al., 2017).

Overall, the proposed model demonstrates the UK housing market characteristics observed in the original (Gilbert et al., 2009). Trends develop when external tweaks to the market are made, showing that indicators are sensitive to these changes in both models. The next stage is to integrate RL, to test whether an intelligent observer agent can learn to identify shocks to the market and deploy countermeasures to minimise their effects in real-time in simulation.

## 5. Results

This section describes the market shock, provides an overview of central bank decisions to deal with shocks, and outlines the healthy housing market conditions. This is followed by a detailed analysis of findings from original housing market research conducted in 2008 (Gilbert et al., 2009) emphasising the experiments we aim to conduct in this article to gauge the strengths and weaknesses of RL. Lastly, experiments and subsequent results show how intelligent RL agents can learn and make decisions in real-time in simulation to counteract induced shocks. Note that actions the RL agent can undertake simplify how decisions are made in the real world. We aim to explore the strengths and weaknesses of this methodology in a simplified housing market, hoping to identify the potential for future applications.

**Figure 5.**
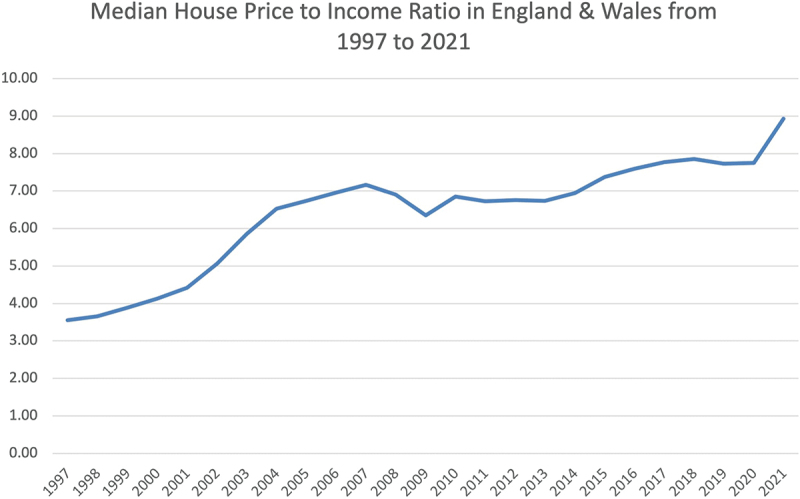
The median house price to income ratio in England and Wales from 1997 to 2021 (source: (Housing Team, 2022)).

Shocks come in various forms, as described in Section 2. To manage shocks, central banks often enforce a monetary policy which stabilises and counteracts the aftermath of the shock (Martin et al., 2022). From 2007 to 2009, the world endured a financial shock resulting in a crash in the UK housing market (Whitehead & Williams, 2011). In Figure 5, the house price to income ratio in England and Wales dropped from 7.17 in 2007 to 6.35 in 2009. Furthermore, a drop of 18.7% in house prices (Munro, 2018), from Q3 2007 to Q1 2009. To counteract the pressures, the central bank reduced interest rates from 5.25 in Jan 2007 to 1.50 in Jan 2009 (Tse et al., 2014). In contrast, a healthy housing market trend may look like Figure 2, where mortgage repayment to income ratio is 20%, and the median house price to income ratio oscillates between 3.5 and 4.0. House prices increase gradually as people move in and out of the market. The number of people in negative equity is as small as possible (Been et al., 2021; Melzer, 2010; Morescalchi et al., 2018).

Gilbert et al. (2009) conducted several experiments using their UK housing market model in 2008, where the model showed how properties of the UK housing market are emergent. Some crucial findings that aligned with empirically observed behaviours of the UK housing market were:

- House price to income ratio showed a stable relationship given mortgage interest rates and the loan-to-value ratio. For example, if interest rates are reduced or loan to value increased, house price to income rises in response.
- When the loan-to-value ratio is 100%, and the market experiences an exogenous interest rate hike from 7% to 10%, a sharp drop in the house price-to-income ratio is observed. However, if loan-to-value is set at 80% and the same increase in the interest rate is observed, the effect is much weaker (Gilbert et al., 2009, p. 5).

These findings were also explored in other housing market research, such as (Narayan & Narayan, 2011; Tse et al., 2014; White, 2015). The experiments (Gilbert et al., 2009) make for a well-documented comparator for this article, where the strengths and weaknesses of RL can be tested in relation to the earlier assertions. Given these original experiments and results, in this article, we expect RL to behave in a certain way when adjusting interest rates in the 100% LTV scenario compared to the 80% scenario. The goal is to test if the RL central bank agent can adapt to these scenarios and fulfil its goals. In the next paragraph, we describe the experiments in detail.

We replicate two experiments; these are “loan-to-value A and B”. In the A experiment, the MaxLoanToValue parameter (refer to Table A1) is 100, and in the B experiment, it is set to 80. In both experiments, an exogenous shock occurs at timestep 200, where mortgage interest rates suddenly increase from 7% to 10%. In the base case experiments (no RL), we observe findings from the (Gilbert et al., 2009). In the RL experiments, we observe behavioural differences and consequences of actions taken by the RL agent in achieving the “healthy housing market” criteria. As our results show, we believe there is value in utilising these contemporary methods to support future research in modelling housing markets.

Given the computational complexity in these models and the nature of RL training, we run the experiments for 100 iterations to capture a distribution of results quantifying model stochasticity. Moreover, during training, we found that the “healthy housing market” criteria were met.

**Figure 6.**
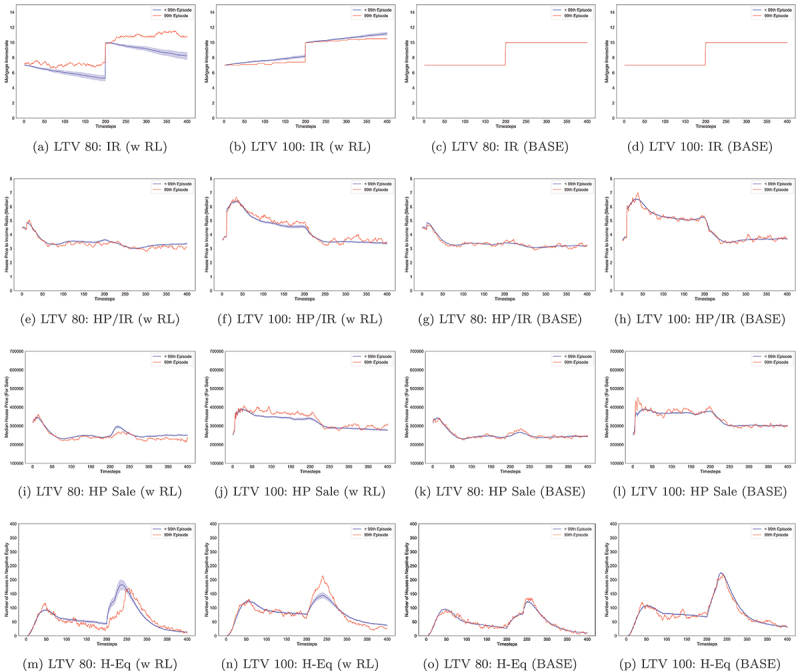
Line graphs showing the last model run ($99^{\mathrm{th}}$ due to index starting at 0) and the average with a confidence interval for all previous runs (<99) aggregated for each experiment condition, including base case conditions. Each row is a tracked variable, and the column is the experiment, where IR = Interest Rates, HP/IR = House Price to Income ratio and H-Eq = Houses in Negative Equity.

To recap, the experiments were run for 400 simulation timesteps. At $200^{\mathrm{th}}$ timestep, a shock impacts key indicators to varying extents as observed in (Gilbert et al., 2009, p. 4) and presented in Figure 6. The ratefall shock severity is greater in 100% LTV compared to 80% LTV; this can be observed in Figures 6(g,h). Similar behaviour is observed for median house prices for sale and the number of households in negative equity (Figures 6(k,l,o,p)).

Interestingly, the RL agent learns to approach the 100% LTV experiment conservatively with slight adjustments but mainly holding interest rates evidenced in Figure 6(b) compared to the 80% experiment Figure 6(a). This is a response to the sensitivity of indicators of the housing market to prevailing interest rates. This particular finding demonstrates the adaptive capabilities of RL, where slight environmental changes in a model can be responded to by learning and experiences. Furthermore, on average the RL agent reduces interest rates for the LTV 80% scenario (see Figure 6(a)), conversely, in the 100% LTV scenario, it increases interest rates on average (see Figure 6(b)).

Another finding from Figure 6 when analysing RL behaviours, in particular, is that leading up to and right after the shock at timestep 200, the confidence intervals are much wider (further from the mean). This means the RL agent has explored more state space at these crucial points during training. This behaviour can be observed most clearly in Figures 6(a,b). In the 100% LTV experiment, the variance statistic for interest rates is 2.691 (mean 9.095, std 1.640) compared to the 80% experiment 4.521 (mean 7.607, std 2.126), where RL agent is exploring more and subsequently adjusting interest rates more often in the 80% compared to the 100% LTV experiment.

RL trains iteratively across several simulation runs, also known as episodes. The latest episode in the training phase is the most recent behavioural output, usually representing the stage at which RL has learned the most optimal set of behaviours to achieve some goal. Conversely, the most recent episodes are those in which RL is yet to learn effective behaviours (Sutton & Barto, 2018). The data observed at the $99^{\mathrm{th}}$ model-run (episode) are the outputs for when the RL was most trained. Therefore, we compare these to our goal criteria for a healthy housing market state. For some indicators, the RL agent was better at achieving the healthy housing market goals than others, which would be expected as some indicators are more sensitive to interest rates than others. For both loan-to-value experiments, RL successfully ensured the house price-to-income ratio was below seven even after the ratefall shock, refer to Figures 6(e,f). The median-house-price-for-sale indicator shows that RL was more effective in achieving the ≤400,000 goal in the 80% experiment (Figure 6(i)) compared to the 100% experiment (Figure 6(j)) which was only above 400,000 for a short time at the earlier timesteps from 0–100. A similar outcome was also observed in the base case Figure 6(l). The data show that the houses-in-negative-equity was a more complex indicator for RL to achieve the goal of ≤123 where the shock exacerbated the complexity as presented by the wide confidence intervals post-shock Figures 6(m,n). However, pre and post-shock, we observe a downward trend where the number of households in negative equity is less than 123, even achieving less than 50 near the end of the simulation. It is worth noting that while differences exist between the RL and base case scenarios, these can be considered small. However, this can result from the chosen healthy housing market goal conditions, and differences may be more significant if other goal conditions or a combination of conditions were chosen. These results also demonstrate the housing market’s ability to settle after a shock.

Overall, these experiments show that RL can achieve healthy housing market goal criteria and alleviate the shock effect on the market, which varies in effectiveness across the different indicators. Given these results, we presume that as the number of goal indicators increases from 3 to 3+i for some i, complexity in achieving these goals also increases. Given this complexity, we believe that RL’s housing market goals may be unachievable at some point. This may be due to equilibrium whereby increasing one indicator, the goal is met, but another indicator is reduced; thus, the goal is not met and vice-versa. This study has demonstrated that RL is a valuable technique that should be welcomed by housing-market and macroeconomic researchers interested in utilising autonomous decision-making methods to aid policy making in dealing with uncertainty like economic shocks. In the next section, we break down the learning process of RL and describe the strengths and weaknesses in utilising RL within this domain.

## 6. Discussion and conclusion

This article reproduces a well-known ABM of the UK housing market to integrate a novel RL algorithm that learns to counteract housing market shocks in real-time. We answer the following research questions: can RL be integrated with housing market ABMs? Moreover, can agents learn trends from the housing market and adapt to economic shocks by counteracting the impact of these shocks in real time? Findings show RL can be integrated with housing market ABMs, as evidenced in sub-Section 3.9 and does well, in learning to counteract shocks through monetary policies such as interest rate adjustments.

This article shows how RL could adapt its behaviours and, over time, through training, learn behaviours that enable it to achieve the goal state. Furthermore, the RL agent portrayed characteristics of the original model (Gilbert et al., 2009). One example is the effects of interest rates in the 80% loan-to-value (LTV) compared to the 100% loan-to-value environment. Responding to the impact of interest rates being more sensitive in the 100% LTV case compared to 80%. The RL agent learned to explore a greater range of interest rates in the less-sensitive scenario (80%) compared to the more sensitive scenario (100%) intended to counteract the market’s sensitivity in these two conditions, which was purely learnt and not hard-coded.

A drawback of our approach is that the RL agent’s tools are limited. This is not indicative of a real-world central bank, which has more policies to counteract crises, such as regulatory, monetary, and fiscal policies. However, macroeconomic policies such as adjusting interest rates is a critical intervention central banks make (Martin et al., 2022; Popescu, 2014; Valadkhani et al., 2019) with the most recent example tackling inflation in the UK (Inman, 2022; Luhnow & Colchester, 2022; Lynch & Adam, 2022). Another caveat is that our model is, a simplified version of the real world. This would ensure computational tractability. Thus, this article only focuses on a single policy tool to demonstrate the application of RL in the housing market research domain and is exploratory in nature.

There are several weaknesses in RL methods, including overfitting, the exploration-exploitation trade-off (Sledge & Principe, 2017), and computational demand. To address the exploration-exploitation issue, we used an objective function that was not greedy but balanced both aspects (Silver et al., 2014; Sutton & Barto, 2018). While computational demand was not a problem for our model, it could become an issue for more complex environments with more agents and action spaces. In these cases, advanced computational resources may be required.

There are several exciting directions for future research based on this work. For example, the findings can support housing market modelling, where researchers forecast the potential for exogenous shocks and identify policy decisions to alleviate economic downturns. The technique can also be adapted to simulate a realistic case where the goal is to optimise the current state of the housing market through RL. Additionally, given the recent release of the 2021 UK census, researchers can enhance the model with this data to study the dynamics of the housing market. This is the first example in the literature that uses novel RL algorithms within housing market agent-based models to develop a methodology for autonomously counteracting exogenous shocks to the market. There is also value in exploring this application in macroeconomics, where artificial intelligence-assisted policy-making and signal detection can have a significant impact. For example, it may help a central bank to detect a recession or financial crisis and take action early.

## Supplementary Material

images.zip

## Notes

The RL model and associated agent-based model (ABM) code can be freely accessed at the following repositories respectively: (Barhate, 2021) and (Olmez, 2022), which includes detailed documentation and instructions for setup and execution. The datasets utilised for training and validating the model are publicly available at https://doi.org/10.6084/m9.figshare.21719879.v1, which includes comprehensive metadata and access guidelines.

## Appendix A.

**Table A1.** Model input parameters and description (source (Gilbert et al., 2009)).

Parameter	Description
InitialVacancyRate	Proportion of empty houses.
nRealtors	Number of realtors.
InterestRate	Mortgage interest rates.
TicksPerYear	Number of timesteps per year.
MaxHomelessPeriod	Maximum number of timesteps a person can be homeless.
InterestPerTick	Interest rate, after a cyclical variation has been applied.
MinPriceFraction	If a house price falls below this fraction of the median price, it is demolished.
Inflation	Inflation percentage per year.
CycleStrength	Percentage of interest rate change periodically.
Affordability	Owners trade down or up depending on the affordability ratio.
Savings	The amount of capital a person has.
ExitRate	Percentage of people exiting the market.
EntryRate	Percentage of people entering the market.
MeanIncome	average annual income.
Shocked	percentage of person’s income increased or decreased.
BuyerSearchLength	Length of time a buyer searches for a home.
RealtorTerritory	The locality a realtor covers.
Locality	Distance between houses used for valuations.
RealtorMemory	Historical collection of records used for valuations.
PriceDropRate	Percentage a house drops in price.
RealtorOptimism	A fee added to the valuation.
InitialGeography	Type of geography.
Density	How clustered the agents are spatially.
HouseConstructionRate	The rate at which houses are constructed.
HouseMeanLifetime	The average lifetime of a house.
MaxLoanToValue	The amount of deposit.
MortgageDuration	The timeframe for mortgage repayment.
Stampduty	A tax levied on house sales (UK only).

**Table A2.** Model input parameters for similarity testing.

Parameter	Input Value
NumberOfRuns	100
Timesteps	400
CycleStrength	0%
MeanIncome	£30000 pa
ExitRate	2%
Density	70%
StampDuty	False
TicksPerYear	4
HouseMeanLifetime	101
MortgageDuration	25
HouseConstructionRate	0.30%
MaxLoanToValue	100%
Shocked	20%
BuyerSearchLength	10
EntryRate	5%
MaxHomelessPeriod	5
InitialGeography	“Random”
PriceDropRate	3%
InterestRate	7%a
RealtorOptimism	3%
Inflation	0%
Affordability	25%
Savings	50%
RealtorTerritory	30
RealtorMemory	10
Locality	3

^a^For scenario two, the interest rate will drop from 7% to 3% initiating a shock at timestep 200.
