# Supplementary figures and images for: Mitigating housing market shocks: an agent-based reinforcement learning approach with implications for real-time decision support
Source: J Simul. 2024 Jul 9;18(6):921–39. doi: 10.1080/17477778.2024.2375446 (PMC11649216; doi:10.1080/17477778.2024.2375446)

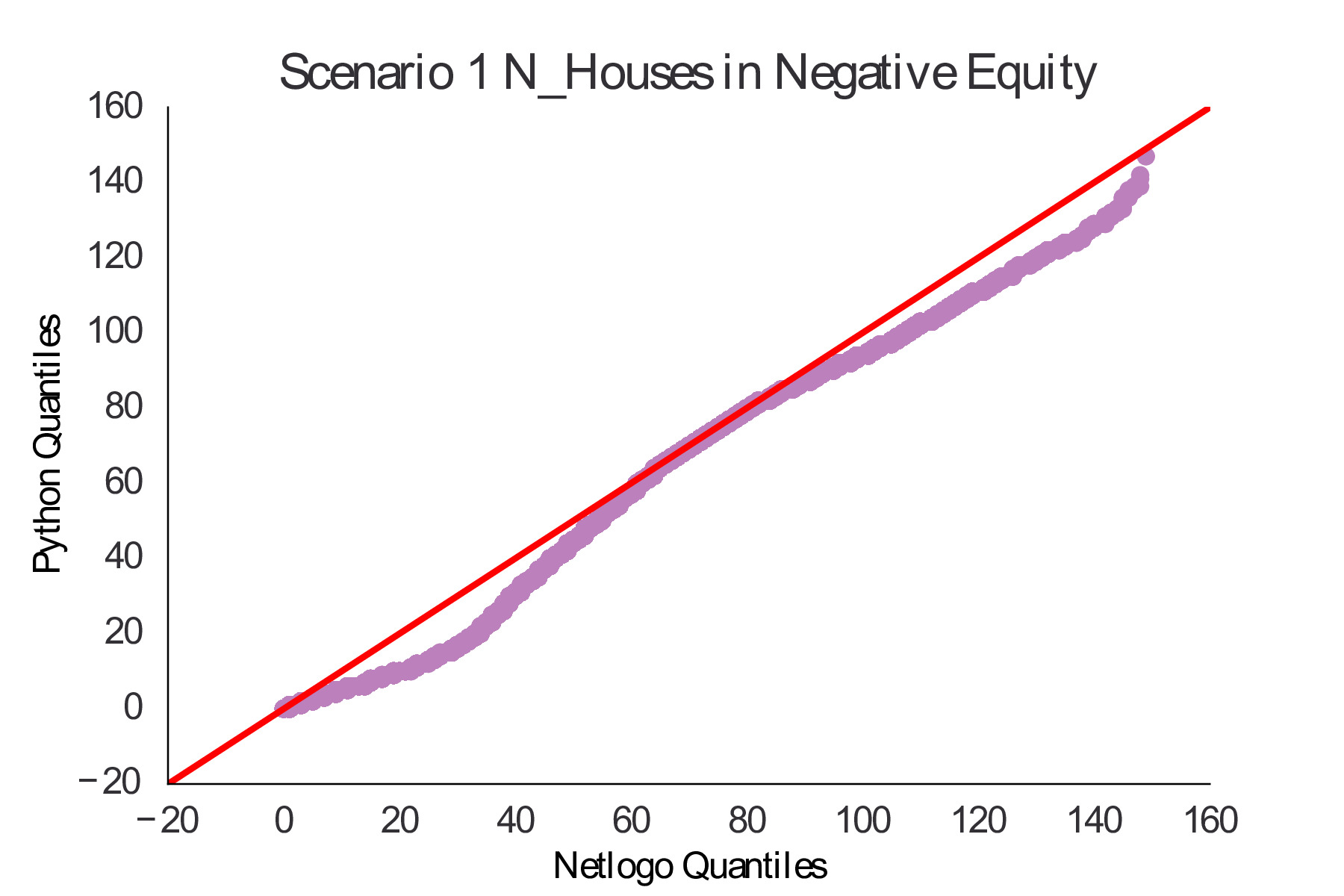

Supplement: images.zip [file TJSM_A_2375446_SM5549.zip › images/QQHouses_in_negative_equity_Scenario1.jpg]

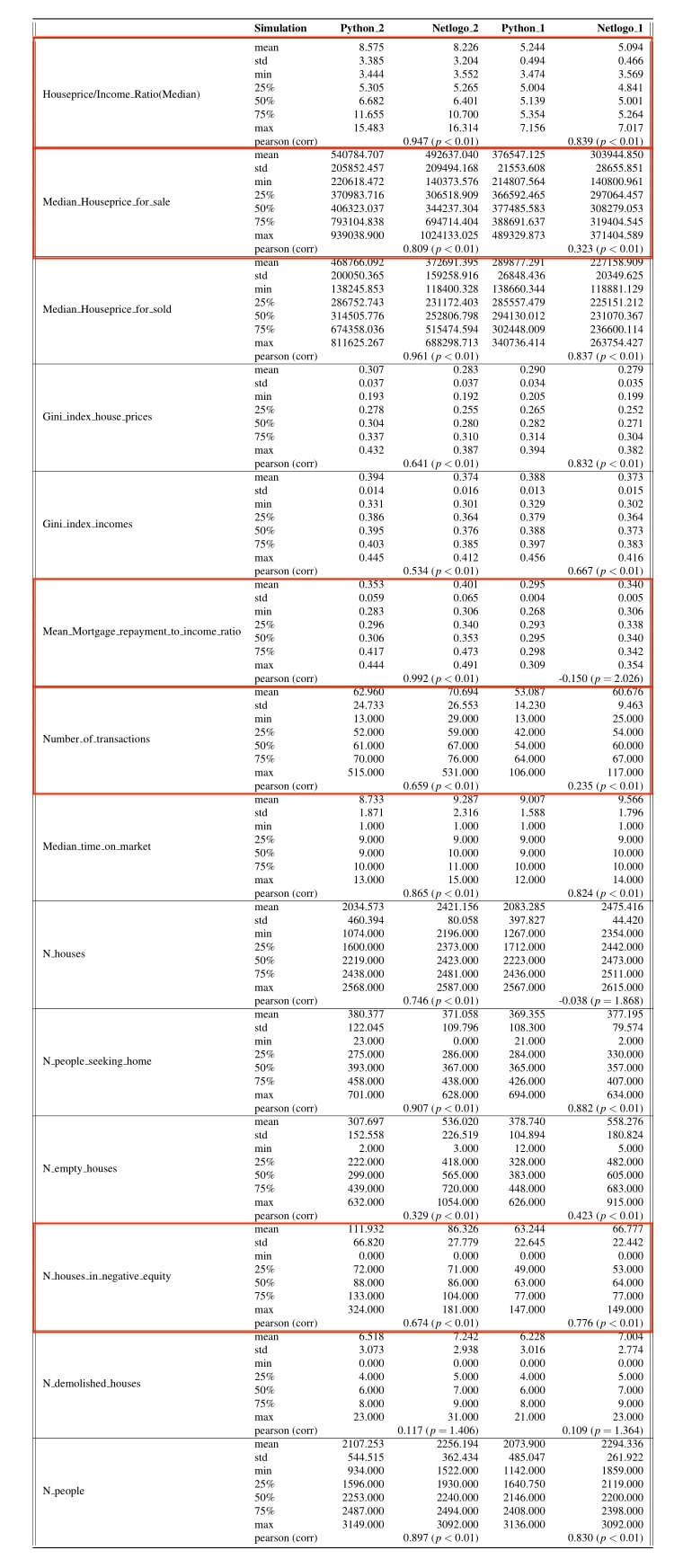

Supplement: images.zip [file TJSM_A_2375446_SM5549.zip › images/table_as_figure_similarities.jpg]

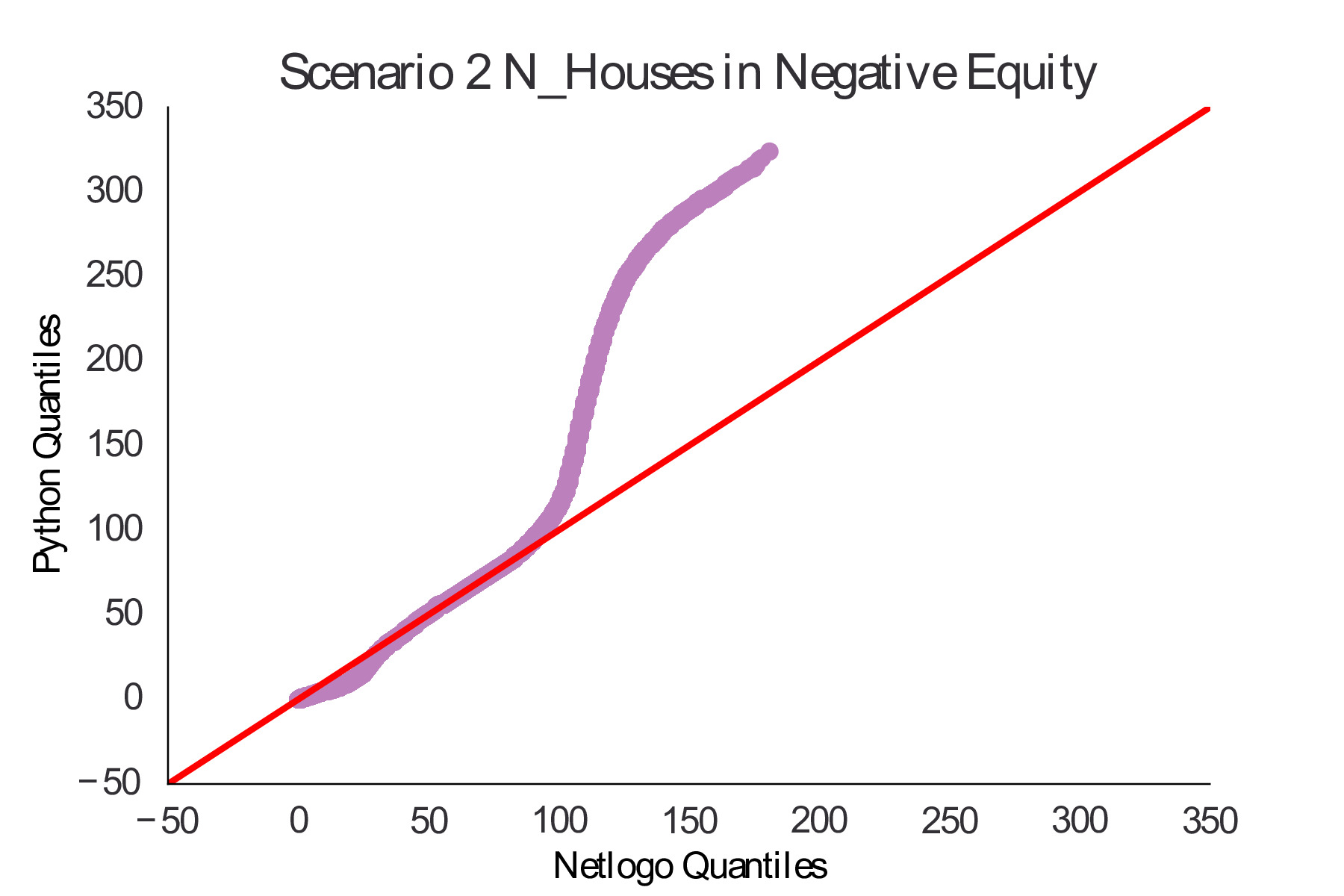

Supplement: images.zip [file TJSM_A_2375446_SM5549.zip › images/QQHouses_in_negative_equity_Scenario2.jpg]

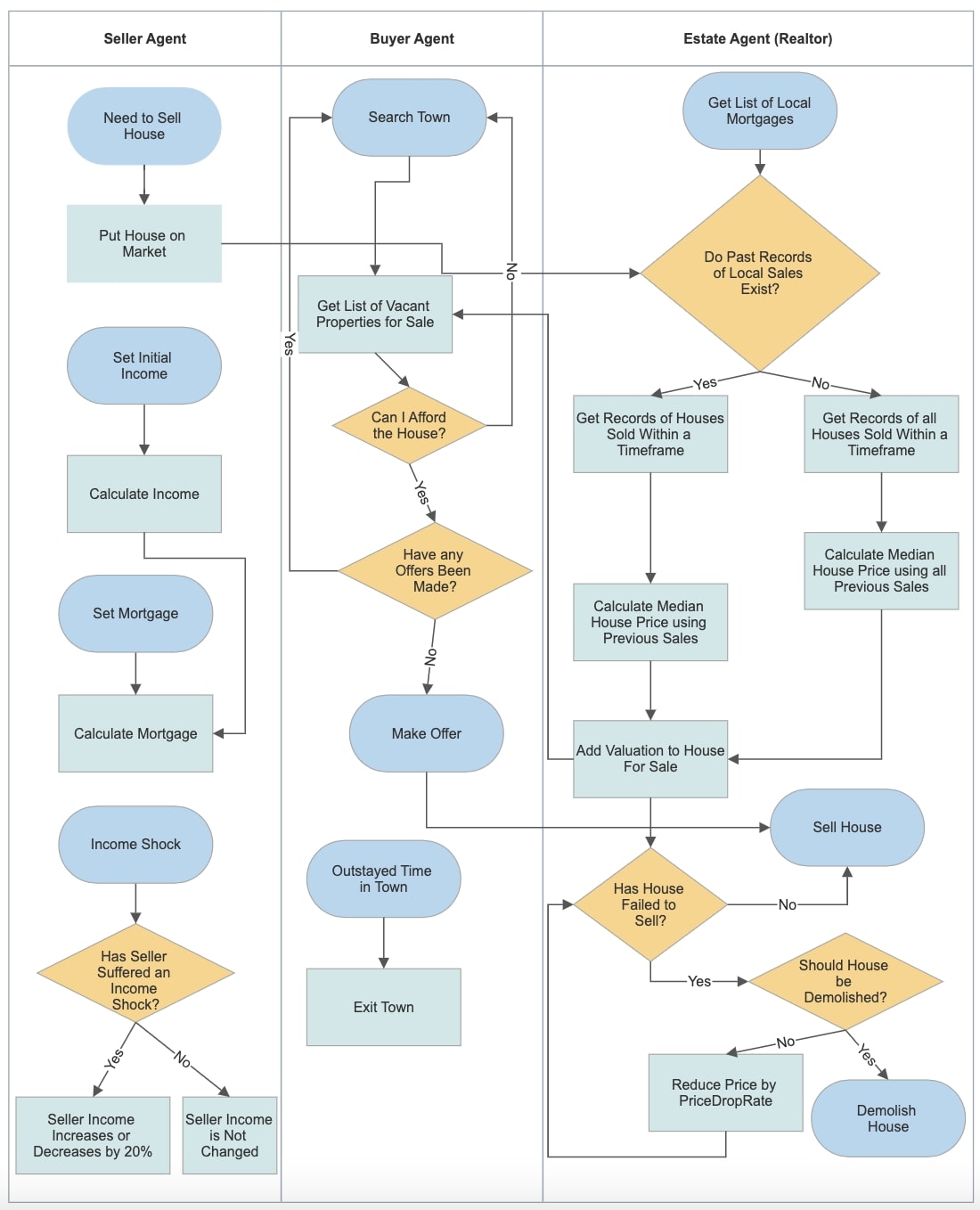

Supplement: images.zip [file TJSM_A_2375446_SM5549.zip › images/flowchart_agent_behaviours.jpg]

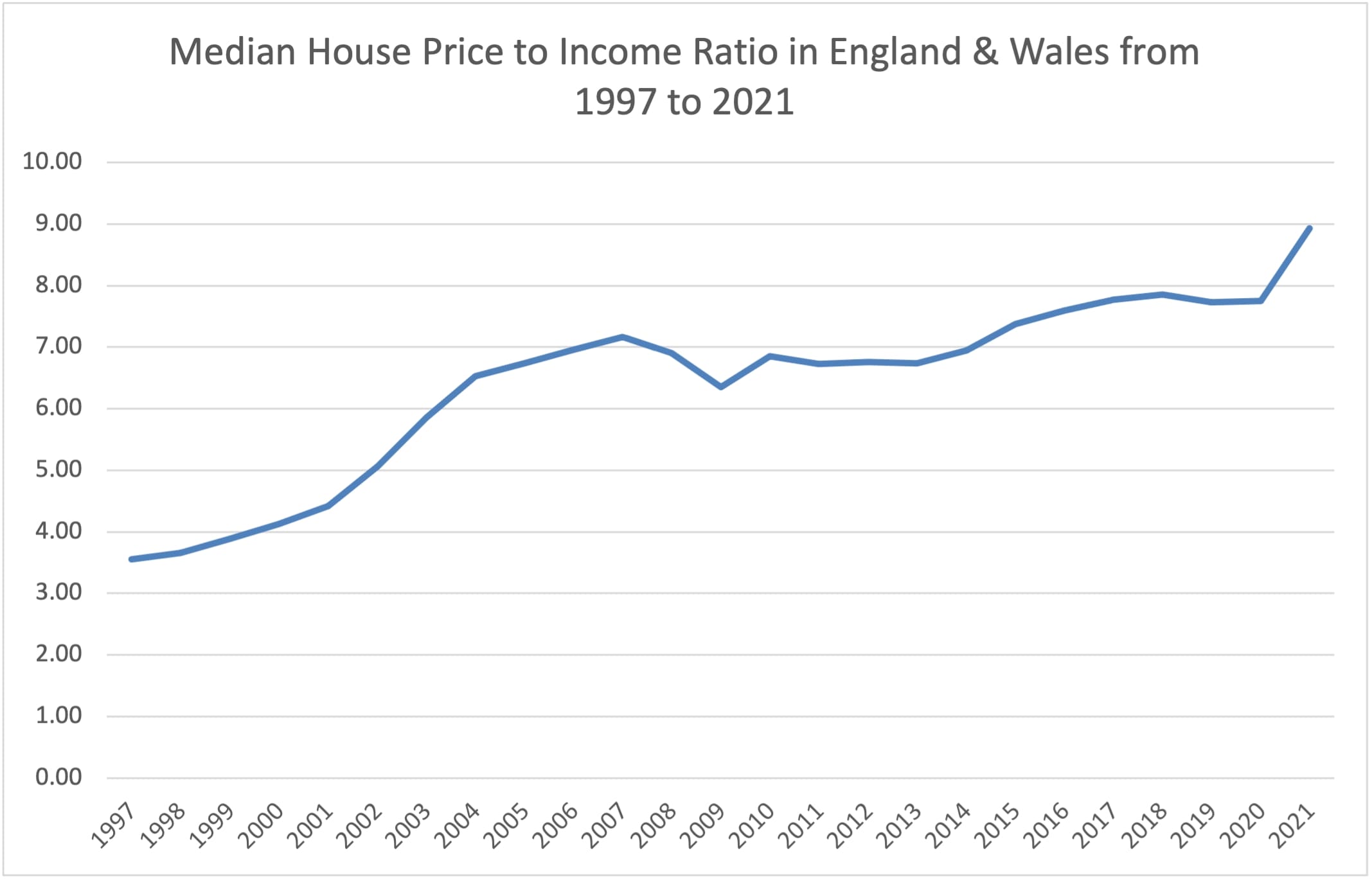

Supplement: images.zip [file TJSM_A_2375446_SM5549.zip › images/MHPIR_Eng_Wls.jpg]

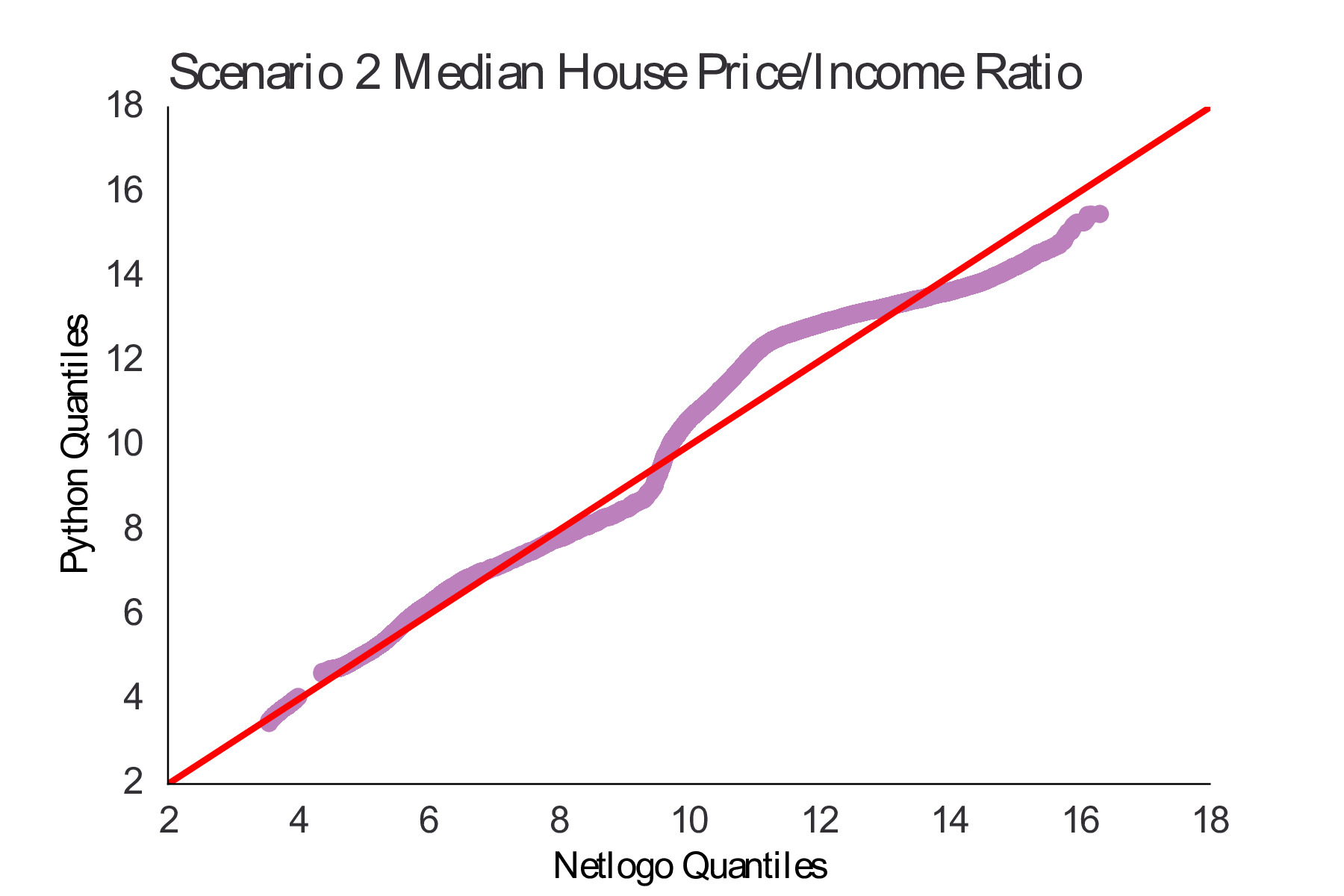

Supplement: images.zip [file TJSM_A_2375446_SM5549.zip › images/QQHP_Income_Scenario2.jpg]

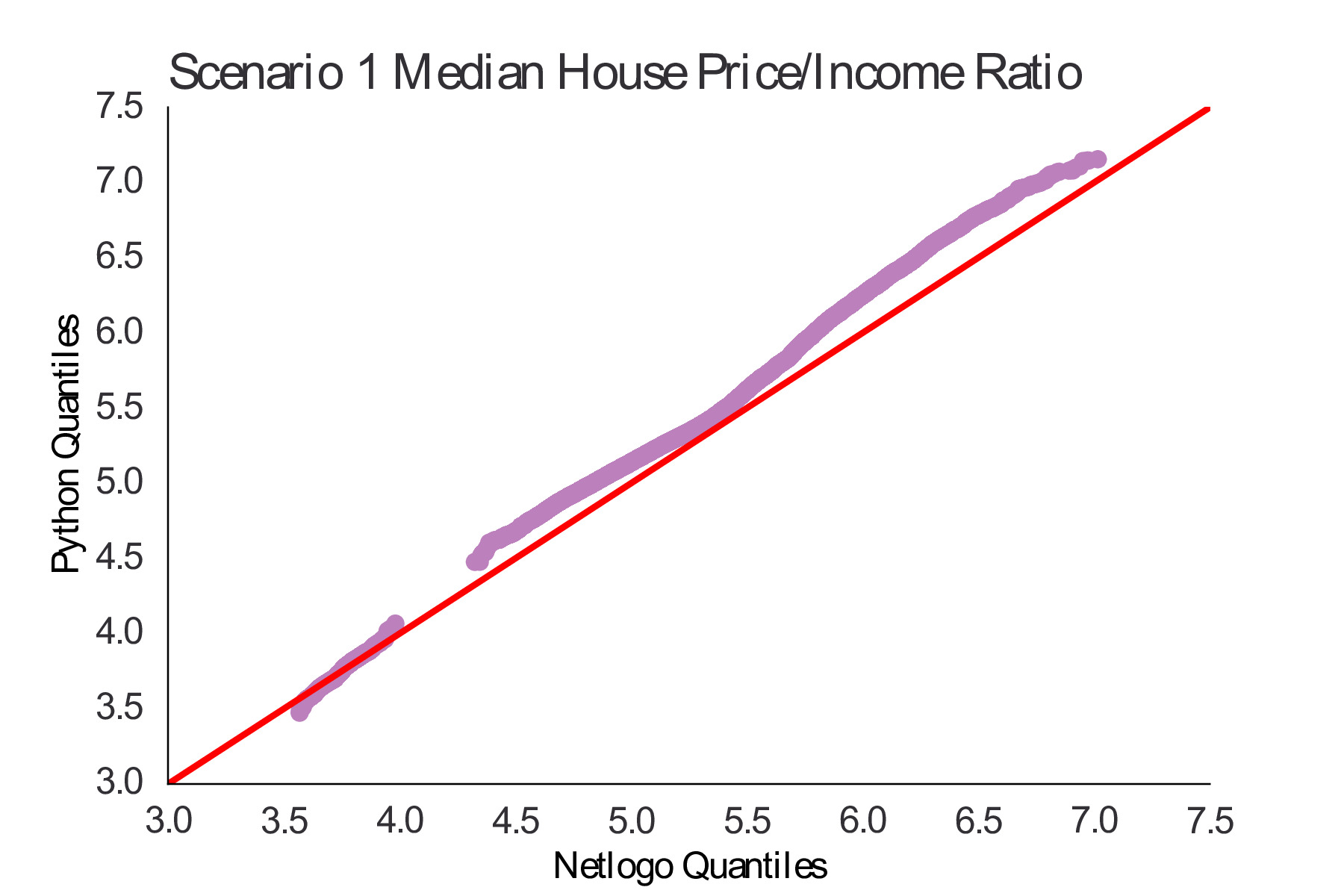

Supplement: images.zip [file TJSM_A_2375446_SM5549.zip › images/QQHP_Income_Scenario1.jpg]

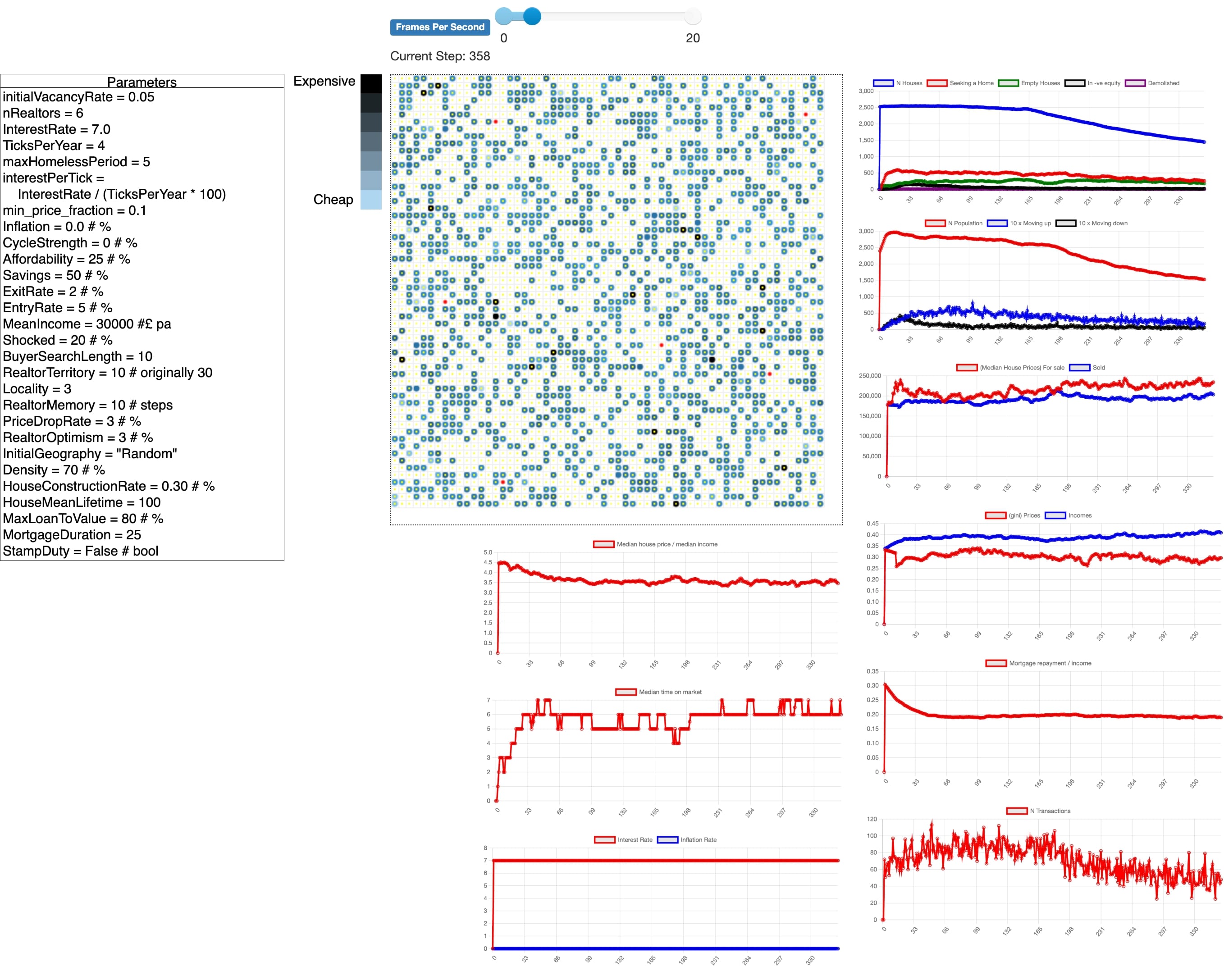

Supplement: images.zip [file TJSM_A_2375446_SM5549.zip › images/model.jpg]

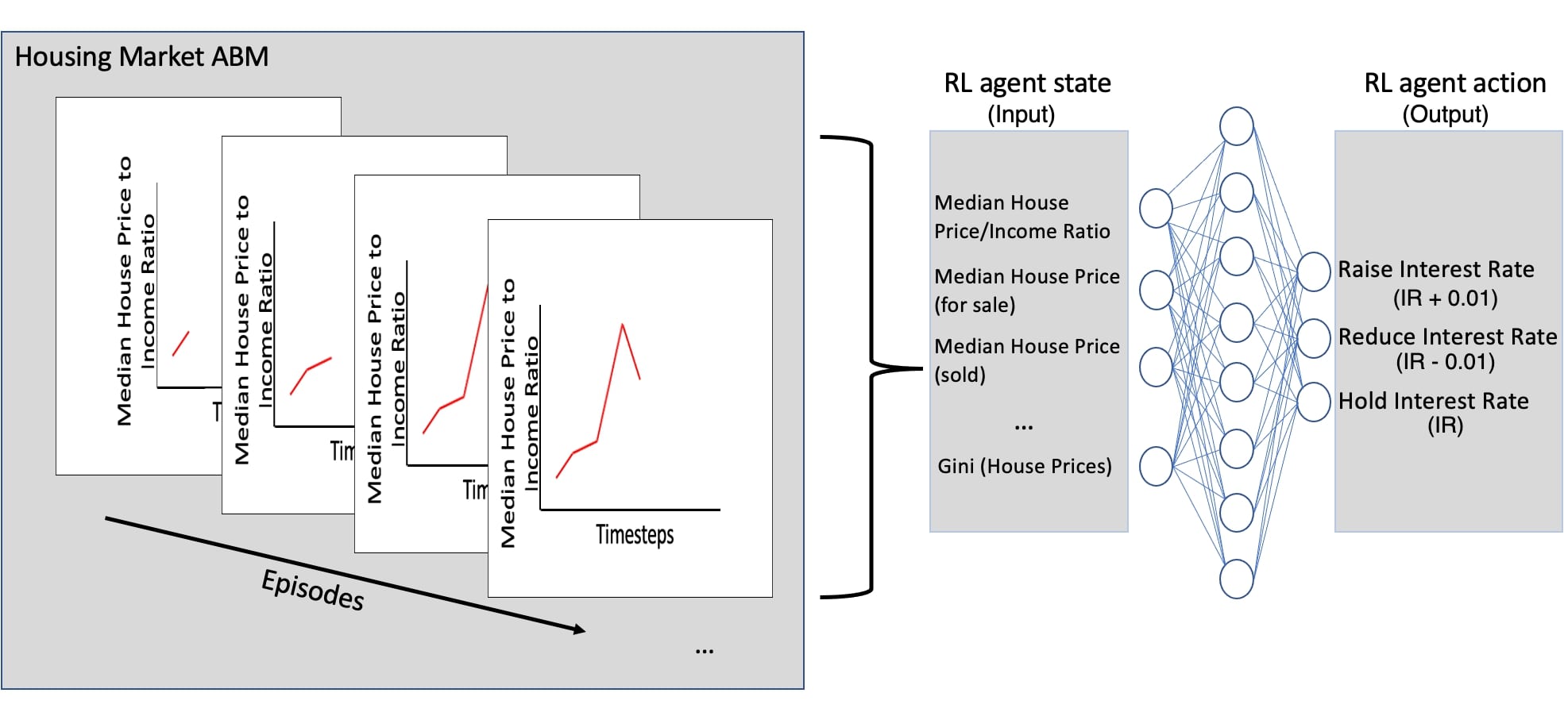

Supplement: images.zip [file TJSM_A_2375446_SM5549.zip › images/RL_agent_diagram.jpg]

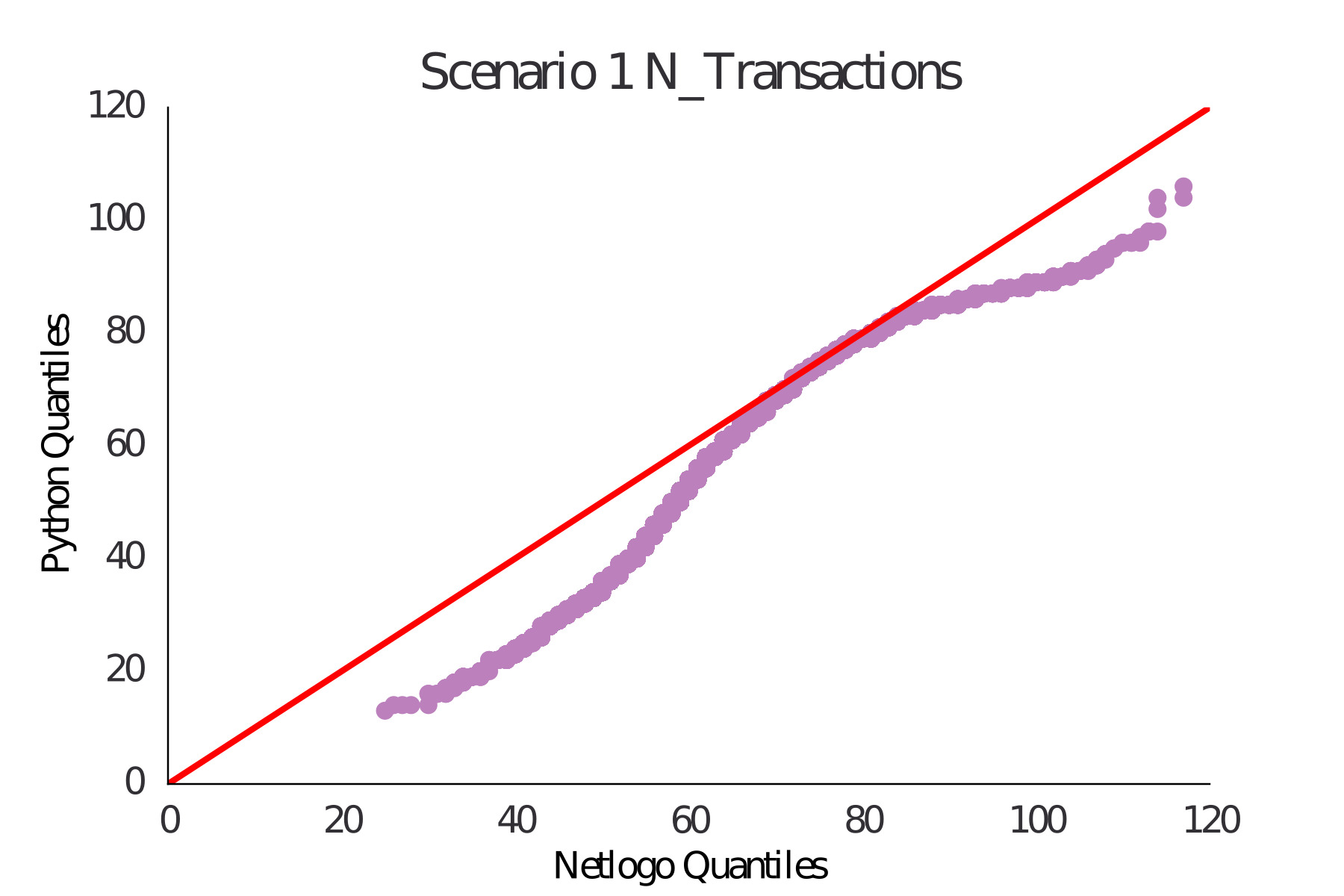

Supplement: images.zip [file TJSM_A_2375446_SM5549.zip › images/QQTransactions_Scenario1.jpg]

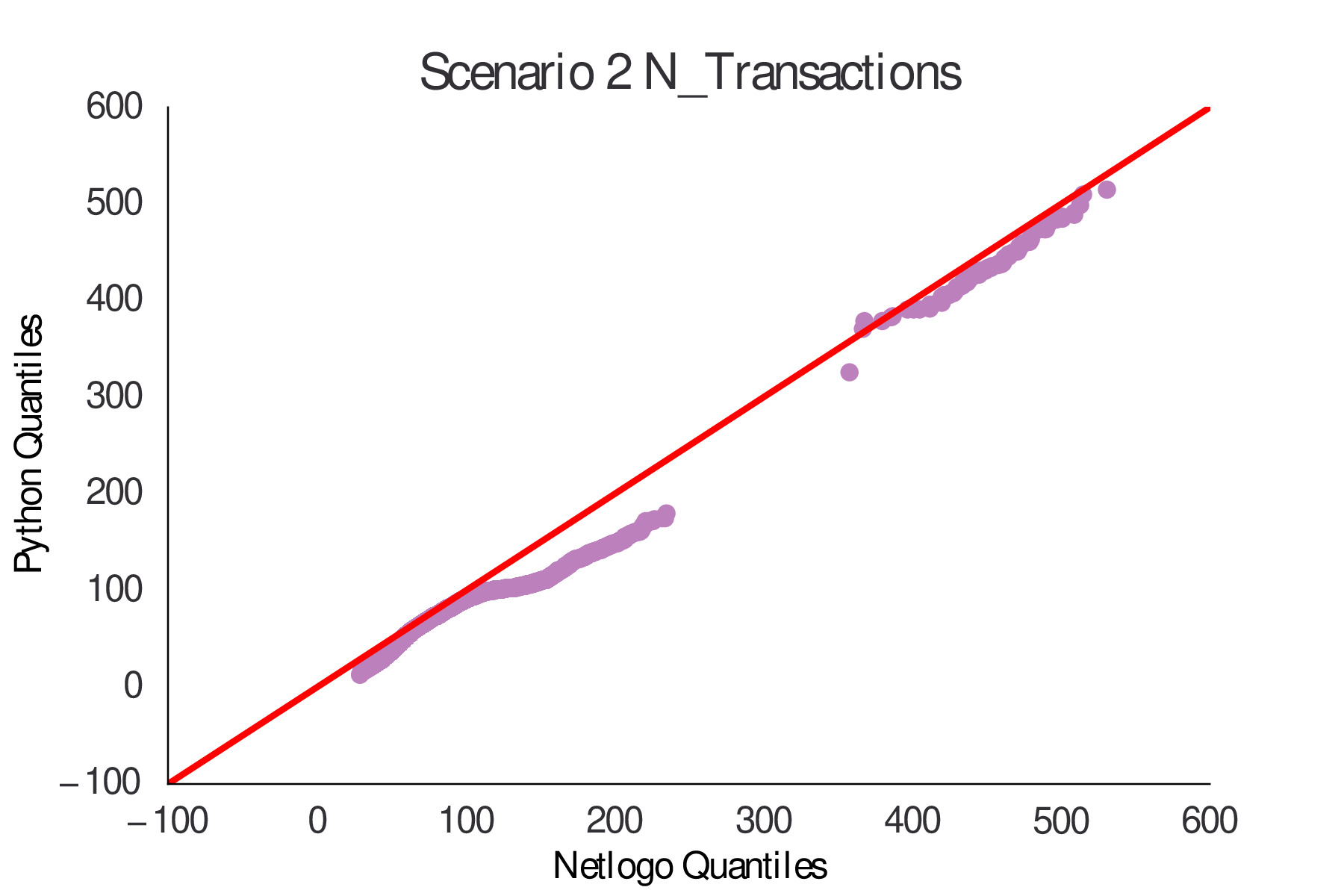

Supplement: images.zip [file TJSM_A_2375446_SM5549.zip › images/QQTransactions_Scenario2.jpg]

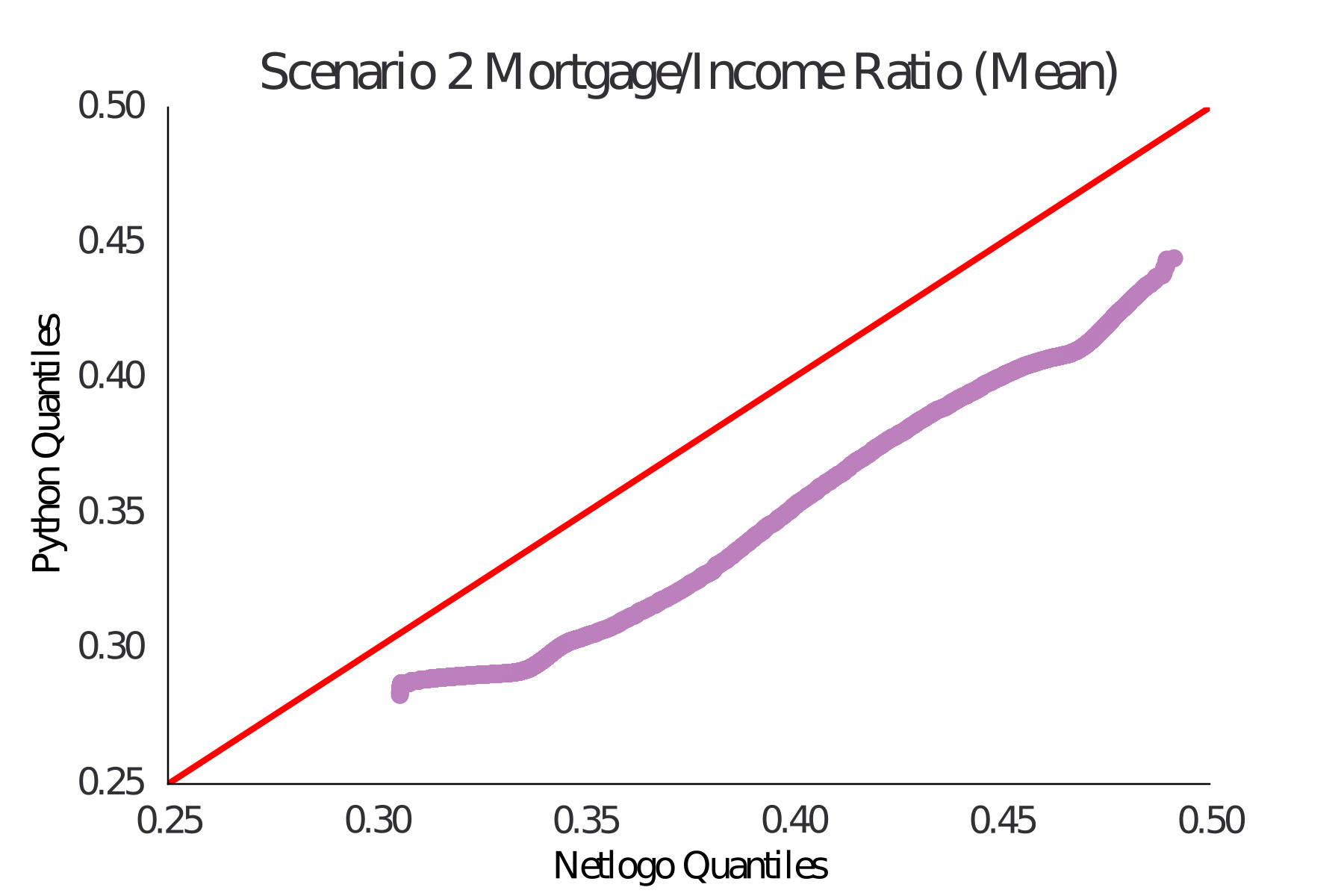

Supplement: images.zip [file TJSM_A_2375446_SM5549.zip › images/QQMortgage_income_Scenario2.jpg]

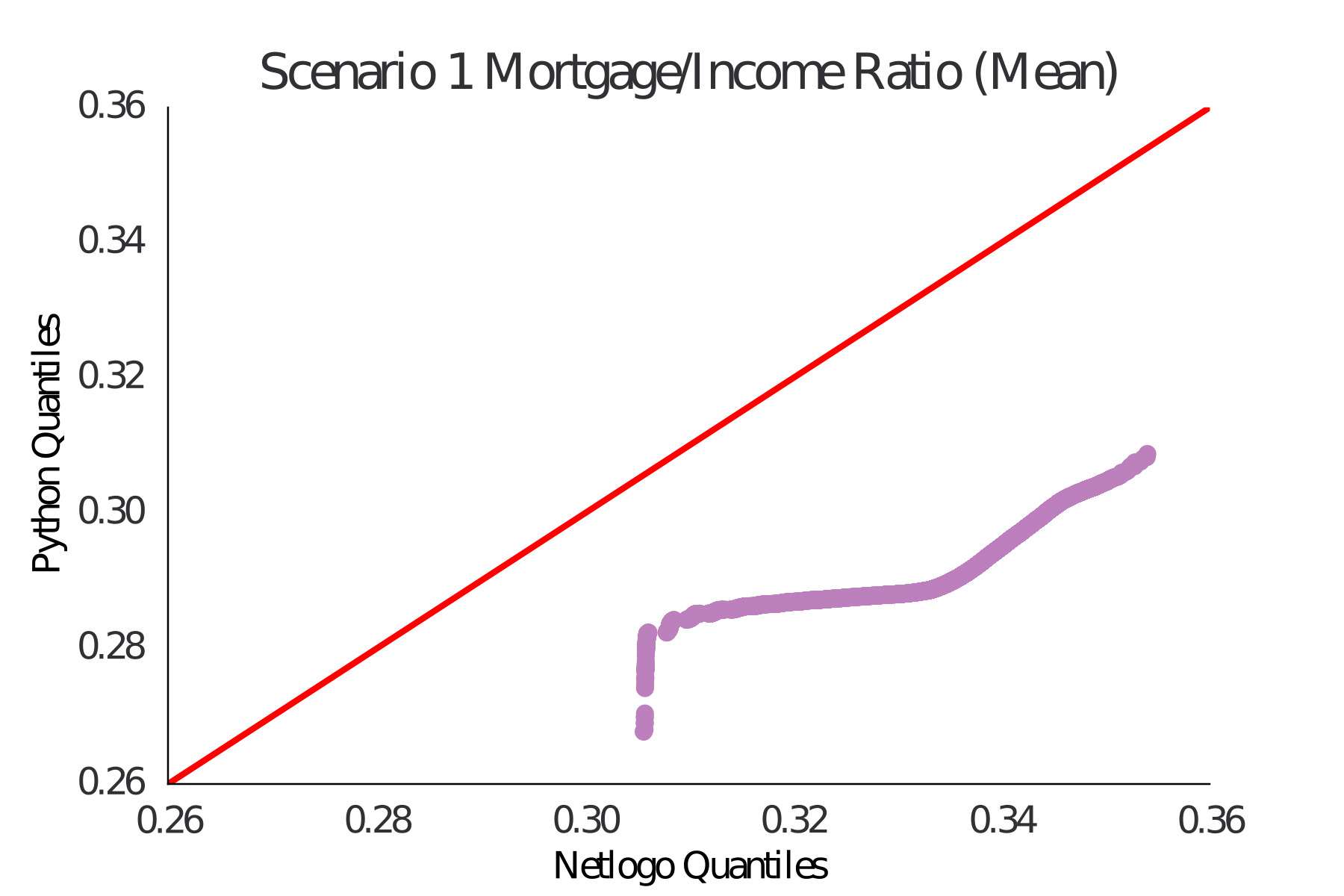

Supplement: images.zip [file TJSM_A_2375446_SM5549.zip › images/QQMortgage_income_Scenario1.jpg]

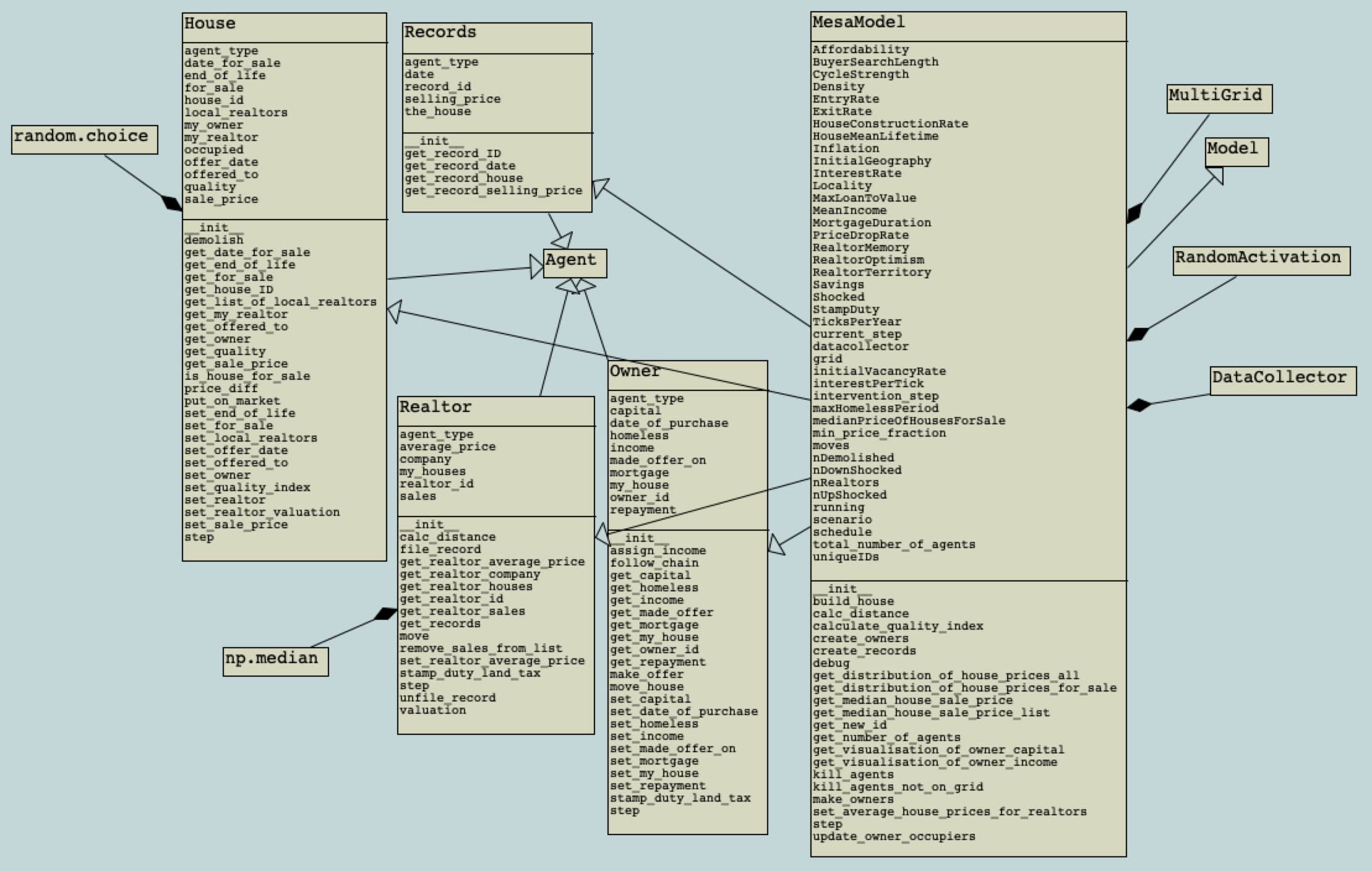

Supplement: images.zip [file TJSM_A_2375446_SM5549.zip › images/class diagram.jpg]

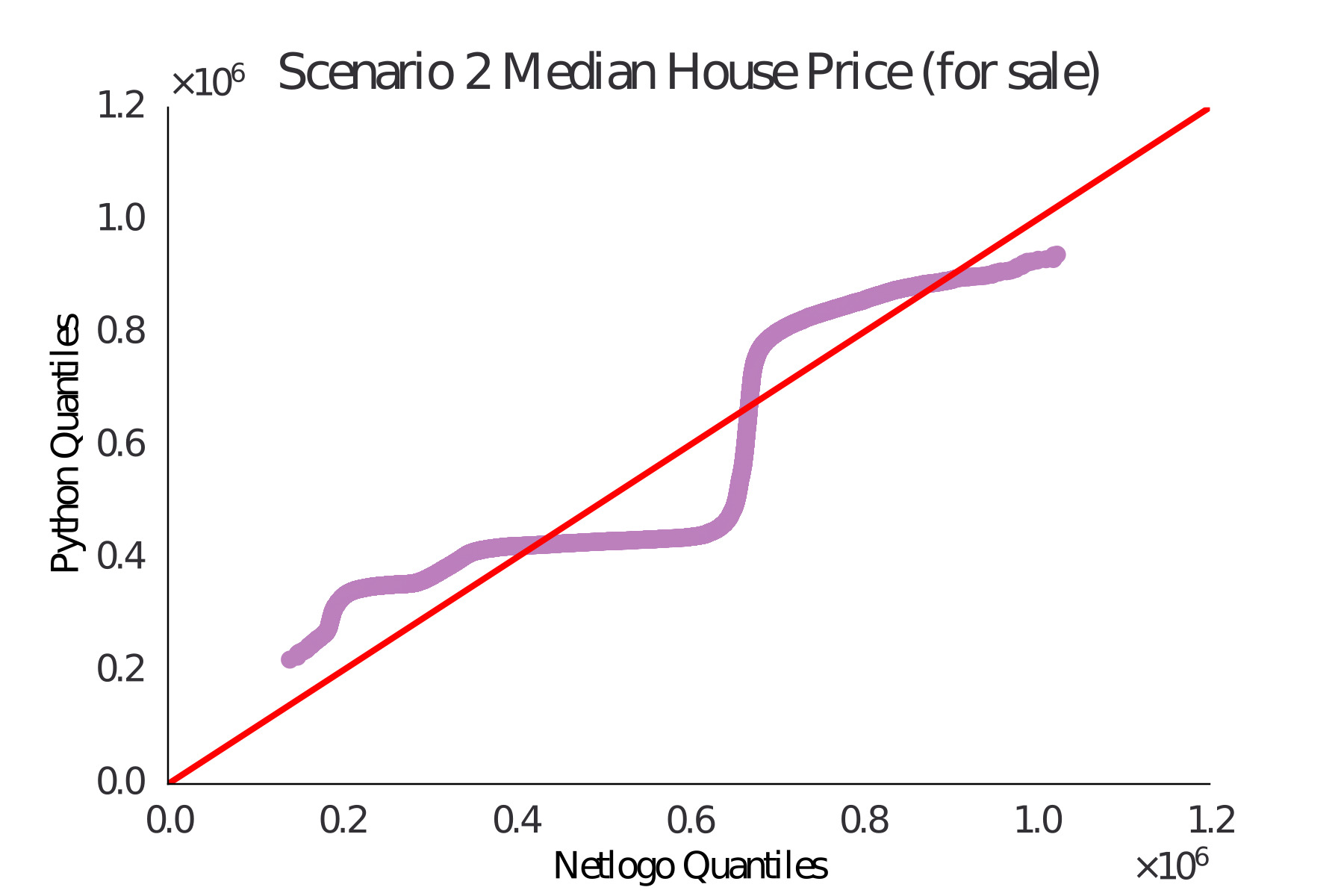

Supplement: images.zip [file TJSM_A_2375446_SM5549.zip › images/QQMedianHP_forsale_Scenario2.jpg]

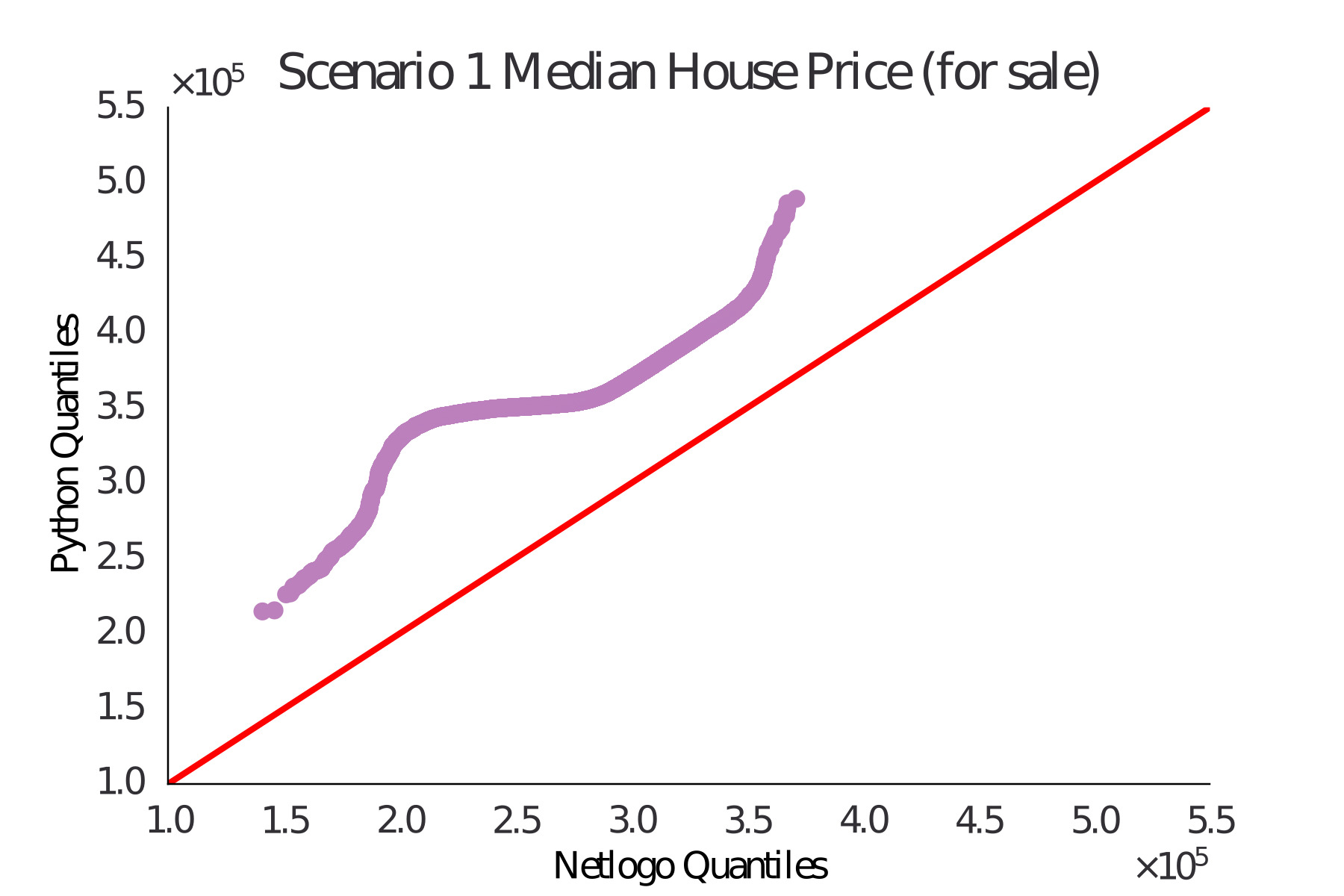

Supplement: images.zip [file TJSM_A_2375446_SM5549.zip › images/QQMedianHP_forsale_Scenario1.jpg]
